# *Bergenia* Genus: Traditional Uses, Phytochemistry and Pharmacology

**DOI:** 10.3390/molecules25235555

**Published:** 2020-11-26

**Authors:** Bhupendra Koul, Arvind Kumar, Dhananjay Yadav, Jun-O. Jin

**Affiliations:** 1 School of Bioengineering and Biosciences, Lovely Professional University, Phagwara 144411, India; 2 Research Center for Chromatography and Mass Spectrometry, CROM-MASS, CIBIMOL-CENIVAM, Industrial University of Santander, Carrera 27, Calle 9, Edificio 45, Bucaramanga 680002, Colombia; arvindtomer81@gmail.com; 3 Department of Medical Biotechnology, Yeungnam University, Gyeongsan 38541, Korea; 4 Research Institute of Cell Culture, Yeungnam University, Gyeongsan 38541, Korea

**Keywords:** *Bergenia* species, botanical description, traditional uses, phytochemistry, pharmacology, anti-urolithiatic activity, bergenin

## Abstract

*Bergenia* (Saxifragaceae) genus is native to central Asia and encompasses 32 known species. Among these, nine are of pharmacological relevance. In the Indian system of traditional medicine (Ayurveda), “Pashanabheda” (stone breaker) is an elite drug formulation obtained from the rhizomes of *B. ligulata*. *Bergenia* species also possess several other biological activities like diuretic, antidiabetic, antitussive, insecticidal, anti-inflammatory, antipyretic, anti-bradykinin, antiviral, antibacterial, antimalarial, hepatoprotective, antiulcer, anticancer, antioxidant, antiobesity, and adaptogenic. This review provides explicit information on the traditional uses, phytochemistry, and pharmacological significance of the genus *Bergenia*. The extant literature concerned was systematically collected from various databases, weblinks, blogs, books, and theses to select 174 references for detailed analysis. To date, 152 chemical constituents have been identified and characterized from the genus *Bergenia* that belong to the chemical classes of polyphenols, phenolic-glycosides, lactones, quinones, sterols, tannins, terpenes, and others. *B. crassifolia* alone possesses 104 bioactive compounds. Meticulous pharmacological and phytochemical studies on *Bergenia* species and its conservation could yield more reliable compounds and products of pharmacological significance for better healthcare.

## 1. Introduction

The use of herbs for healing diseases and disorders can be dated back to at least 1500 BC [1]. The traditional system of medicine (TCM) is a source of >60% of the commercialized drugs and is still used by the population in lower income countries for the cure of chronic diseases [2]. As far as primary healthcare is concerned, approximately 75% of Indians rely on Ayurvedic formulations [3,4]. Many medicinal plants containing various phytochemicals have been successfully used to cure diabetes, cancers, gastrointestinal disorders, cardiovascular, and urological disorders [1].

Among the urological disorders, “urolithiasis” is the third most common disorder with a high relapse rate [5,6,7,8]. The invasive treatments of urolithiasis are costly and precarious, so the search for natural anti-urolithiatic drugs is of immense importance [9,10].

The Ayurvedic preparations have used *Bergenia* species down the centuries to dissolve bladder and kidney stones and to treat piles, abnormal leucorrhea, and pulmonary infections [11,12,13]. These pharmacological properties can be attributed to a wide-range polyphenols, flavonoids, and quinones present in *Bergenia* species. The polyphenols constitute a major share of the active ingredients, and the elite among them are ”arbutin” and “bergenin” [14,15,16,17,18,19]. Bergenin alone possesses burn-wound healing, antiulcer, anti-arrhythmic, antihepatotoxic, neuroprotective, antifungal, antidiabetic, antilithiatic, anti-inflammatory, anti-nociceptive, anti-HIV, and immunomodulatory properties [20,21,22]. *Bergenia ligulata* Wall. Engl. [synonym of *B. pacumbis*] is an essential ingredient of an Ayurvedic formulation, “Pashanbheda” (Paashan = rockstone, bheda = piercing), which is used as a kidney stone dissolver in the indigenous system of medicine [23,24]. This drug has been listed in ancient Indian chronicles of medicine including “*Charak Samhita*”, “*Sushruta Samhita*” and “*Ashtang-Hridaya*”. *B. ligulata* is reputedly known by other names such as “Pashana”, “Ashmabhid”, “Ashmabhed”, “Asmaribheda”, “Nagabhid”, “Parwatbhed”, “Upalbhedak”, and “Shilabhed” [25].

The unavailability of a compendious review on bioactive molecules present in *Bergenia* genus prompted us to compile the same. The present review provides explicit knowledge on the traditional and medicinal importance and phytochemistry of the *Bergenia* species.

## 2. Review Methodology

The extant literature (abstracts, blogs, full-text articles, PhD theses, and books) on the *Bergenia* species was reviewed systematically to generate concise and resourceful information regarding their distribution, phytochemistry, traditional medicinal uses, and pharmacological activities. For this purpose, different bibliographic search engines and online databases (Google Scholar, WoS, PubMed, CAB abstracts, INMEDPLAN, Scopus, NATTS, EMBASE, SciFinder, MEDLINE) and websites (www.sciencedirect.com; eflora.org; jstor.org; pfaf.org) were referred, to select 174 references for detailed analysis. Each botanical name has been validated through www.theplantlist.org and https://www.catalogueoflife.org/ online repositories. ChemDraw software (version 12.0) was used to draw the structures of the chemical compounds.

## 3. Distribution

**Figure 1:**
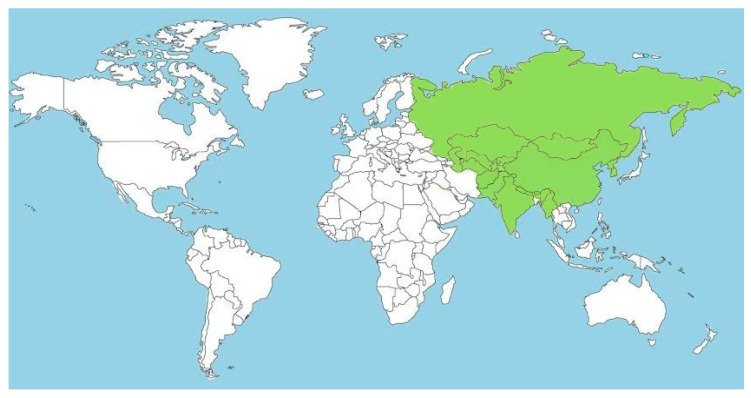
A world map showing the geographical distribution of *Bergenia* species (in green).

The plant family Saxifragaceae encompasses 48 genera and 775 species, which are mostly distributed in South East Asia. The name “Bergenia” was coined by Conrad Moench in 1794, in the memory of Karl August von Bergen (German botanist and physician). Genus *Bergenia* harbors 32 species of flowering plants, including highly valued ornamental, rhizomatous, and temperate medicinal herbs [16]. Central Asia is the native place for genus *Bergenia* [26,27]. The geographical distribution of 32 species of genus *Bergenia* are detailed in Figure 1, which depicts the worldwide distribution through the map. In China, seven species are reported from three provinces and two autonomous regions: Shanxi, Sichuan, and Sanxi and Tibet and Xinjiang, respectively. Among the seven species, four (*B. yunnanensis*, *B. scopulosa*, *B. emeiensis*, and *B. tianquanensis*) are endemic to China [28,29,30].

## 4. Botanical Description

*Bergenia*(s) are evergreen, perennial, drought-resistant, herbaceous plants that bear pink flowers produced in a cyme. Due to the leaf shape and leathery texture, *Bergenia*(s) have earned some interesting nicknames such as “pigsqueak”, “elephant-ear”, “heartleaf”, “leather cabbage”, or “picnic plates”. The plants should be planted about two feet apart as they spread horizontally up to 45–60 cm. The botanical description of *Bergenia* species [31,32,33,34] is described in Supplementary Table S1.

## 5. Traditional Medicinal Uses

*Bergenia* species have been used in the traditional medicines for a long time. In Unani and Ayurvedic systems of medicine, *Bergenia* spp. rhizomes and roots have been used for curing kidney and, bladder diseases, dysuria, heart diseases, lung and liver diseases, spleen enlargement, tumors, ulcers, piles, dysentery, menorrhagia, hydrophobia, biliousness, eyesores, cough, and fever [35,36,37]. The burns or wounds may be treated with rhizome paste for three to four days [38,39,40]. The paste can be applied on dislocated bones after setting, or consumed to treat diarrhea or along with honey in fevers [41,42].

The leaf extract of *B. ciliata* possesses antimalarial property [43]. Its leaves are revered to as “Pashanabheda”, which designates the litholytic property [44]. In Nepal, 1:1 mixture (one teaspoon) of the dried B. ciliata rhizome-juice and honey is administered to post-partum women 2–3 times a day as a tonic and remedy for digestive disorders (carminative) [38]. The rhizome-decoction may also be consumed orally as antipyretic and antihelmintic [45].

Since ancient times, consumption of water-extract of *B. ligulata* has cured urogenital and kidney-stone complaints [23,35,46,47]. In Nepal, the rhizome paste of *B. ligulata* is consumed for treating many diseases including diarrhea, ulcer, dysuria, spleen enlargement, pulmonary infusion, cold, cough, and fever [45]. The intestinal worms can also be removed by consuming rhizomes along with molasses (two times/day, 3–4 days) [38]. The Indians use the dried roots of *B. ligulata* for treating burns, boils, wounds, and ophthalmia [46,48]. The dried leaf powder of *B. pacumbis* may be inhaled to bring relief from heavy sneezing [49]. In Lahul (Punjab), the locals use *B. stratecheyi* plants to prepare a poultice, which is applied to heal the joint-stiffness [50]. *Bergenia* species are also used for the treatment of boils and even blisters [19].

In Russian tradition, *B. crassifolia* leaves are commonly used to prepare a health drink. Buryats and Mongols used *B. crassifolia*-young leaves of to prepare tea. Interestingly, in Altai, tea is prepared from old blackened leaves (chagirsky tea having lesser amounts of tannins) [51]. The rhizome infusions can treat fevers, cold, headache, gastritis, dysentery, and enterocolitis [52]. They are also used to treat oral diseases (bleeding gums, periodontitis, gingivitis, and stomatitis) and also possess adaptogenic properties [51,53,54,55]. Mongols used the extracts for treating typhoid, gastro-intestinal ailments, diarrhoea, and lung inflammation. The rhizome extract is also used to strengthen capillary walls to stop bleeding after abortions, alleviate excessive menstruation, and cervical erosion. Therefore, the roots and rhizomes of *B. crassifolia* are claimed as antimicrobial, anti-inflammatory, haemostatic, and as astringent in the officinal medicine of Mongolia [54].

Tibetans apply fresh leaf-paste on their skin to protect them from harmful ultraviolet radiations [56]. The chewing of leaf helps in relieving constipation and the leaf-juice can treat earaches [11,38,42]. The bullocks and cows are fed on a mixture of *Bergenia* inflorescence and barley-flour to treat hematuria [38]. *Bergenia* roots are also effective in preventing venereal diseases [57]. Thick leaves of Bergenias are used in Chinese Medicine to stop bleeding, treat cough, dizziness, hemoptysis, and asthma, and to strengthen immunity [27,58].

## 6. Phytochemistry

Nowadays, HPLC and HPTLC have become routine analytical techniques due to their reliability in quantitation of analytes at the micro or even nanogram levels plus the cost effectiveness. Phytochemical investigation of nine *Bergenia* species (*B. ciliata*, *B. crassifolia*, *B. emeiensis*, *B. ligulata*, *B. scopulosa*, *B. stracheyi*, *B. hissarica*, *B. purpurascens*, and *B. tianquanesis*) led to the characterization of several chemical constituents [16,59,60,61,62,63]. The review of the extant literature reveals the presence of 152 chemical compounds (volatile: 47 and non-volatile: 105) (Table 1) as shown in Supplementary Figure S1. The constituents have been categorized into polyphenols, flavonoids, quinones, sterols, terpenes, tannins, lactones, and others [16,26,64,65,66,67]. The major bioactive compounds are bergenin (**1**), (+)-catechin (**2**), gallic acid (**3**), β-sitosterol (**4**), catechin-7-*O*-*β*-d-glucoside (**5**), (+)-afzelechin (**6**), *arbutin (**10**),* 4-*O*-galloylbergenin (**12**), 11-*O*-galloylbergenin (**13**), caffeoylquinic acid (**21**), pashaanolactone (**26**), 3,11-di-*O*-galloylbergenin (**64**), bergapten (**66**), kaempferol-3-*O*-rutinoside (**70**), quercetin-3-*O*-rutinoside (**79**), (+)-catechin-3-*O*-gallate (**83**), 2-*O*-caffeoylarbutin **(86)**, leucocyanidin (**124**), methyl gallate (gallicin) (**125**), sitoinoside I (**126**), *β*-sitosterol-d-glucoside (**127**), avicularin (**128**), reynoutrin (**129**), procyanidin B1 (**135**), afzelin (**140**), and aloe-emodin (**146**).

Arbutin (**10**) inhibits tyrosinase, prevents the formation of melanin and thus prevents skin darkening [68]. Bergenin (**1**) is a pharmaceutically important molecule that has hepatoprotective and immunomodulatory potential [69]. It is used clinically for eliminating phlegm, relieving cough, inflammation, etc. [20,70,71]. (+)-catechin (**2**) possesses antioxidant, glucosidase, renoprotective, matrix-metalloproteinase inhibitory, and cancer preventive activity. Gallicin (**125**) exhibits antioxidant, anti-tumor, antimicrobial, anti-inflammatory, and cyclooxygenase-2/5-lipoxygenase inhibitory activity [72]. Gallic acid (**3**) possesses anti-inflammatory, antioxidant, cytotoxic, bactericidal, gastroprotective, and antiangiogenic activity. *β*-sitosterol (**4**) is well-known for its antioxidant, anti-inflammatory, analgesic, and anti-helminthic effects. It is also efficient in the curing prostate enlargement [73].

Recently, bergenicin (**151**) and bergelin (**152**) have been isolated from leaves of *B. himalaica* Boriss [71]. The chemistry of *B. tianquanesis* plant has not been reported to date. Although several bioactive compounds have been isolated and characterized from *Bergenia* species, there is still scope for extended research on their efficacy and versatility.

## 7. Pharmacological Activities

**Figure 2:**
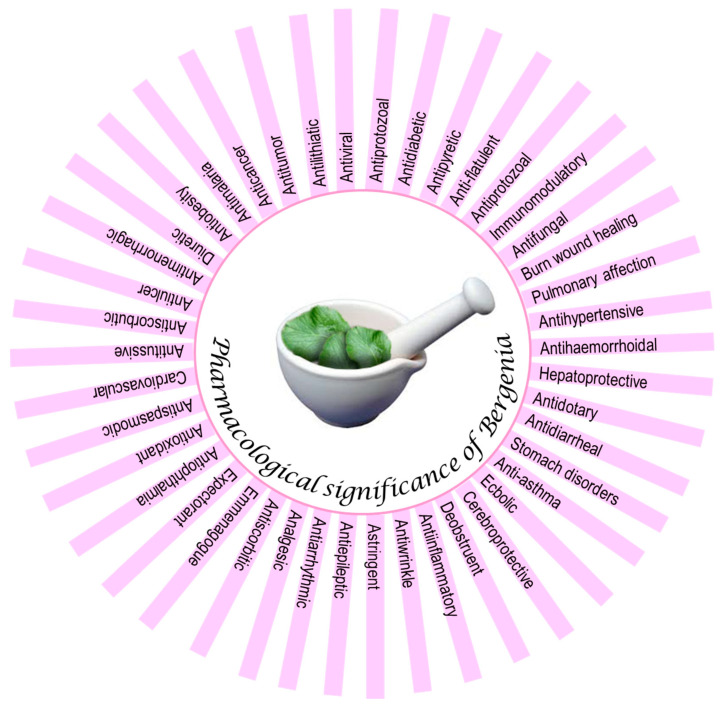
Pharmacological significance of *Bergenia* species.

The pharmaceutical importance of *Bergenia* species has been known since ancient times. Therefore, numerous biopharmaceutical products encompassing leaf or stem extracts are available in the markets and are being used to cure specific ailments (Figure 2).

### 7.1. Antilithiatic Activity

The major contribution of *B. ligulata* towards pharmaceutical applications is that of an antilithiatic agent. Lower dose (0.5 mg/kg) of the EtOH extract of *B. ligulata* rhizome encourages diuresis in rats and is effective in dissolving preformed stones [144]. The MeOH extracts of the rhizome also possess an antilitihiatic property that has been tested both in vitro and in vivo. In male Wistar rats, 5–10 mg/kg of the extract inhibited calcium oxalate crystal (CaC_2_O_4_•_x_) aggregation in the renal tubes. There are several other reports that state that *Bergenia* extracts exerts its antilithiactic effect by diuresis, inhibition of CaC_2_O_4_•_x_ crystal formation and aggregation, and hypermagnesemic and antioxidant activity [106,145,146].

### 7.2. Diuretic Activity

*Bergenia* species are also known to possess diuretic properties. The EtOH extracts of *B. ligulata* roots were tested for their diuretic activity in rats. The Na^+^, K^+^, and Cl^−^ ion concentrations and the volume of urine excreted was measured after an interval of 5 h. It was observed that the EtOH extract showed significant diuretic activity [107]. *Bergenia crassifolia* (L.) Fritsch. leaf extract contains 15–20% arbutin, which has the potential to treat genitourinary diseases. In a 14 day experiment, the rats were injected with arbutin (**10**) and hydroquinone (**89**), 5 mg/kg (seven days) and 15 mg/kg (seven days). During the experiment, the arbutin (**10**) treatment increased the urine output (diuresis) along with creatinine and potassium, while hydroquinone (**89**) did not [147].

### 7.3. Antidiabetic Activity

After rigorus researches on animal models, it has now been proved that *B. ciliata*, *B. ligulata,* and *B. himalaica* possess an antidiabetic property [71]. The EtOH extracts of *B. ligulata* roots exhibit a remarkable hypoglycaemic effect in diabetic rats [108]. Saijyo et al., (2008) isolated the antidiabetic principle (*α*-glucosidase inhibitor) from *B. ligulata* rhizome extract by column chromatography, which was characterized as (+)-afzelechin (**6**), by NMR technique [61]. The antidiabetic property of *B. ligulata* can be useful in developing nutraceuticals (value-added food products) for diabetics [61,71,108].

### 7.4. Antitussive Activity

*Bergenia* species possesses the potential antitussive property. Different concentrations of arbutin (**10**) were administered to cough-induced mice, and it was observed that a dose of 200 mg/kg had the similar effect as that of 30 mg/kg antitussive drug codeine phosphate [138].

### 7.5. Insecticidal Activity

It has been recently discovered that *B. ligulata* exhibit an insecticidal property. The volatile oil from roots of *B. ligulata* containing 1,8-cineole (**119**) [4.24%], (+)-(6*S*)-isovaleric acid (**120**) [6.25%], (+)-(6*S*)-parasorbic acid (**121**) [47.45%], terpinen-4-ol (**122**) [2.96%], and (*Z*)-asarone (**123**) [3.50%] was tested for its insecticidal activity against *Drosophila melanogaster*, which was found to be significant [109]. Thus, volatile oil from *Bergenia* species or its specific component could be deployed as a natural insecticidal agent [24,109].

### 7.6. Anti-Inflammatory Activity

*Bergenia* species do have anti-inflammatory potential. The aqueous and EtOH (50%) extract of the rhizomes were introduced to animal model (rats) to demonstrate the anti-inflammatory activity. The succinate dehydrogenase (SDH) activity level (represented higher in inflammation) reduced in the rats that received the therapy. The attenuation of inflammatory response was confirmed through pharmacological and biochemical measurements [148]. Different concentrations of the MeOH extract of *B. ciliata* rhizomes have also been tested on a rat model with 100 mg/kg phenylbutazone (an anti-inflammatory agent) as a standard. Maximum inhibition of the inflammatory response was recorded at a dose of 300 mg/kg [74]. In a study by Churin et al. (2005), the dry extract of *B. crassifolia* leaves was administered to DBA/2 mice to study the effect on immune response. The extract declined the inflammatory process by preventing T-lymphocyte accumulation and cytokine production in the inflammatory region [149].

In another study, the delayed type hypersensitivity reaction was significantly elevated in mice administered with 100 μg/mL of bergenan BC (pectic polysaccharide) extracted from *B. crassifolia* leaves. It enhanced the uptake volume of neutrophils and mediated oxygen radicals’ production by mouse peritoneal macrophages [150]. In mice model (balb/c mice), the increasing dose of bergenin (**1**) extracted from the rhizomes of *B. stracheyi* exhibited anti-arthritic property in a dose-dependent manner up to a dose of 40 mg/kg, while a higher dose of 80 mg/kg caused a reduction in the same [151]. These studies along with several others explain the anti-inflammatory activity of *Bergenia* species [92,110,151].

### 7.7. Antipyretic Activity

*B. ligulata* possess a significant antipyretic property. In a study by Singh et al. (2009b), the EtOH (95%) and aqueous extract of *B. ligulata* prepared in 2% gum acacia was administered to Wistar rats (300 and 500 mg/kg body weight) having pyrexia [107]. The antipyretic activity was observed using 200 mg/kg paracetamol (standard antipyretic drug) as positive control. The rectal temperature of the rats was documented after the 1 h time interval. A significant lowering in the body temperature was observed with EtOH extract (500 mg/kg). This study along with others justify that *B. ligulata* possesses significant antipyretic potential [111].

### 7.8. Anti-Bradykinin Activity

The anti-bradykinin activity of *B. crassifolia* leaf extract (per oral dose/treatment: 50 mg/kg for 14 days) has been studied in *spontaneously hypertensive* (SHR) rats. The reduction in the systolic blood pressure was observed after 3–6 h (by 20–25 mmHg), while a lowering of diastolic blood pressure with similar values was observed after 1 h of treatment [112,152]. The angiotensin-I-converting enzyme converts the hormone angiotensin I to the active form (vasoconstrictor: angiotensin II) and thus indirectly elevates the blood pressure by causing the blood vessels to constrict. The EtOH (70%) extract of *B. crassifolia* rhizomes significantly inhibits the angiotensin-I-converting enzyme (IC_50_ = 0.128 mg/mL), in vitro, and thus exhibits anti-bradykinin activity [153].

### 7.9. Antiviral Activity

The MeOH-water extract from rhizomes of *B. ligulata* have been reported to impede the in vitro replication of influenza A virus. Pre-treatment of cells with *B. ligulata* extract was effective in the preventing virus-mediated cell-destruction by repressing viral RNA and protein synthesis. The aqueous extract of *B. crassifolia* leaf supplemented with lectins reduced the virus-induced (HSV strain L2) cytopathogenic effect up to 95% [55]. The bioactive compound 1,2,3,4,6-penta-*O*-galloyl-*β*-d-glucose (**133**) present in the EtOH extract of *Saxifraga melanocentra* Franch. has been tested for its antiviral activity against HCV NS3 serine protease, through ELISA. The IC_50_ values of penta- (**133**), tetra- (**84**) and 2,4,6-tri-galloyl-β-d-glucose (**23**), were estimated to be 0.68–1.01 μM and exhibited 98.7–94.7% inhibition [113,128]. 1,2,3,4,6-penta-*O*-galloyl-β-d-glucose and its derivatives are also reported in *Bergenia* species. Thus, the aforementioned results support the antiviral potential in *Bergenia* species also.

### 7.10. Antibacterial Activity

Almost all of the aforementioned nine *Bergenia* species possess antibacterial activity. In a study by Sajad et al. (2010), the antibacterial activity of *B. ligulata* whole plant extract was analyzed based on the diffusion method. Different concentrations (10, 25, or 50 mg/mL) of the aqueous, EtOH and MeOH extracts of *B. ligulata* rhizomes exhibited antibacterial activity against *E. coli*, *B. subtilis*, and *S. Aureus* [110]. The extract concentration of 50 mg/mL was found to be most effective and was similar to that of the ciprofloxacin-antibiotic (25 µg/mL). These results show that *B. ligulata* possess significant antibacterial activity [110]. It is reported for *B. ciliata* that compared to leaf extracts, the root and rhizome extracts exhibit much higher antibacterial activity. The MeOH rhizome extracts of *B. scopulosa* were tested on eight different bacteria(s) using the agar-well diffusion assay method. It was concluded from the bacterial susceptibility test that both Gram-ve and +ve bacteria are susceptible as evident from the zone of inhibition that ranged from 13 to 15 mm. However, *E. coli*, *P. aeruginosa*, *K. pneumoniae*, and *S. aureus* were found to be vulnerable, as they were considerably inhibited at a concentration of 12.5 mg/mL [129]. In a similar study, the *B. scopulosa* MeOH extract was tested for its inhibitory effect on *S. aureus*, *P. aeruginosa*, and *E. coli*, through zone-inhibition assay. It was interesting to note that the inhibitory impact on *S. aureus* was stronger than that on *P. aeruginosa* and *E. coli* [134].

### 7.11. Antimalarial Activity

Malaria is a notorious disease and one of the main causes of high morbidity and mortality in many tropical and subtropical areas. The ethnopharmacological relevance of the *Bergenia* species for treating fever has been time-tested. EtOH leaf extracts *B. ciliata* (ELEBC) has been tested for its antiplasmodial (*Plasmodium berghei*) activity using a rodent-malaria model, along with chloroquine (10 μM) as a positive control. The IC_50_ of ELEBC was found to be less than 10 μg/mL. Thus, both the in vitro and in vivo experiments have confirmed the antimalarial activity of ELEBC [43].

### 7.12. Hepatoprotective Activity

*Bergenia* species do possess hepatoprotective potential. In a study, the EtOH root-extract of *B. ligulata* was evaluated for its hepatoprotective activity in CCl_4_ treated (toxicant) albino rats. The estimation of hepatoprotective activity was confirmed by measuring the decline in the elevated levels of serum marker-enzymes such as SGPT, SGOT, ALP, and total bilirubin levels [107]. In another study conducted by Mansoor et al. (2015), the *B. ligulata* leaf extract (dose of 500 mg/kg) fully restored the carbon tetrachloride (potent hepatotoxicant)-induced variations in carbon tetrachloride intoxicated rats [154]. Moreover, the histopathological examination of the liver tissue further confirmed the hepatoprotective effect [154]. *B. crassifolia* dry extract has also been reported to exhibit hepatoprotective property in rats intoxicated with 4-pentenoic acid, thus confirming its hepatoprotective potential [155].

### 7.13. Antiulcer Activity

In some areas of South East Asia, *B. ciliata* has been used in the treatment of stomach disorders as a folkloric medicine. An experiment was performed to assess the gastro-protective activity of *B. ciliata* extracts on stomach ulcer-induced rats. Different doses (15, 30, and 60 mg/kg) of the aqueous and MeOH rhizome extracts were administered 1 h after the ulcerogenic treatment. Among the two treatments, the aqueous extract reduced the stomach-ulcer lesions to a better degree. It was concluded that the rhizome extract exhibited its cytoprotective effect (anti-ulcer activity) by facilitating the improvement of gastric mucosal barrier [75].

### 7.14. Anticancer Activity

*Bergenia ciliata* rhizome extracts (MeOH and aqueous) were tested for their cytotoxicity on human breast, liver, and prostate cancer cell-lines by XTT assay, respectively. Both the extracts exhibited concentration-dependent toxicity in each of three cell lines [156]. The IC_50_ value of both extracts fell within the acceptable range in all cell-lines (except Hep 3B cell-lines). Thus, Bergenias possess potential antineoplastic activity that may have probable clinical use as preventive medicine [76,77].

### 7.15. Antioxidant Activity

Undoubtedly, *Bergenia* species are an excellent source of antioxidants. *B*. *ciliata* MeOH leaf extract has been reported to be a potent free-radical scavenger (EC_50_ of 36.24 μg/mL), as confirmed through DPPH assay [78,157]. *B. ligulata* also possess considerable antioxidant activity, as confirmed by DPPH assay (IC_50_ value: 50 µg/mL) [93]. Ivanov et al. (2011) reported that the antioxidant properties of *B. crassifolia* is due to the presence of two compounds, (+)-catechin-3,5-di-*O*-gallate (**82**) and (+)-catechin-3-*O*-gallate (**83**). They were isolated from its aqueous EtOH leaf extract and exhibited strong antioxidant properties, as determined by DPPH assay, with SC_50_ = 1.04 and 1.33 g/mL, respectively [72].

Shilova et al. (2006) performed a study using green and black leaves EtOH extracts of *B. crassifolia* and examined the oxygen uptake rate in a gasometric system with 2,2′-azobisisobutyronitrile-initiated oxidation of isopropylbenzene. The green leaves showed the most pronounced antioxidant effect [158]. In another study, the separation of main phenolic compounds of *B. crassifolia* followed by their DPPH assay with the post-chromatographic derivatization of TLC plates. The increasing order of the free-radical scavenging activity was found to be gallic acid > arbutin > ellagic acid > hydroquinone > ascorbic acid [94]. A comparative assessment of the antioxidant activity, free radical scavenging activity, and inhibition of lipid-peroxidation using MeOH and aqueous extracts of *B. ciliata* rhizomes was performed. The MeOH extract exhibited a better antioxidant activity [76].

### 7.16. Antiobesity Activity

It was reported by Ivanov et al. (2011) that crude extracts of *B. crassifolia* rhizomes can efficiently suppress the human pancreatic lipase activity (IC_50_ = 3.4 g/mL) in vitro [72]. The *B. crassifolia* leaf extracts are known to suppress the appetite as well as energy intake in rats suffering from high-calorie diet-induced obesity. Compared to controls, a 40% reduction in the daily dietary consumption of the rats tested with 50 mg/kg *Bergenia* aqueous leaf extract (seven days of oral treatment) was observed. Moreover, a reasonable reduction (45%) in the triglyceride level was also observed after seven-day therapy [159]. 3,11-Di-*O*-galloylbergenin (**64**), a galloylbergenin from *B. crassifolia* roots has been reported (using MC3T3-G2/PA6 murine preadipocytes) to exhibit a moderate anti-lipid accumulation activity [160].

### 7.17. Adaptogenic Activity

An adaptogen increases the resistance power against various stresses such as physical, chemical, or biological stress and has a stabilizing effect on the body functions [161]. *B. crassifolia* can also be considered as a promising phytoadaptogen [53,55]. In a treadmill test, the running-time of rats fed (for 10 days) on 300 mg/kg *Bergenia* black leaves extract was elevated by 30% more than the control group. The running-time was similar to that of rats administered with 5 mL/kg of extract of *Eleutherococcus senticosus* [162]. Similarly, the swimming capacity of the mice treated with infusions prepared from *B. crassifolia* fermented leaves was observed to significantly increase by 2.2-fold, compared to the control. The swimming capacity was increased with a simultaneous increase in glucose utilization and without changing the body weight [163]. A similar study revealed that the endurance capability of rats exposed to a very low temperature of −15 °C (3 h, for 21 days) was significantly ameliorated after treatment with extracts of *Bergenia* black-leaves. Moreover, the floating-time of the rats supplemented with 100 mg/kg extract was considerably augmented after 21 days of treatment, whereas in the other group treated with liposome-encapsulated-extract the swimming-time was increased after seven days of treatment, under extreme circumstances (e.g., hypoxia) [164], because, under hypoxic conditions, the adaptive response of an organism activates mitoK_ATP_ channel and increases the ATP-dependent potassium transport in mitochondria. Mironova et al. explored the the activation ability of mitoK_ATP_ channel through water-soluble flavonoid-containing plant preparations of *Bergenia (Bergenia crassifolia)* in a rat model [165].

## 8. Other Benefits of *Bergenia* Species

Bergenias are a reservoir of nutrients and are therefore used in culinary preparations [63]. Furthermore, the arbutin **(10)** content of Bergenias inhibits the degradation of insulin and is useful for diuresis and can work as a urinary disinfectant [56]. Bergenias are also being used in the field of cosmetics, owing to the presence of arbutin [166]. The arbutin can make skin whiten because it can prevent tyrosinase activity and can reduce the skin’s melanin (pigment) production [14,167]. *B. ligulata* is used for manufacturing cosmetic brightening agents and under-eye creams [23]. *B. emeinensis* extracts have also been used to treat skin wrinkles [168].

## 9. Conclusions and Future Perspectives

It is quite evident from this review that the *Bergenia* species contains a wide range of bioactive compounds of therapeutic value. The safety and efficacy of *Bergenia* leaves and rhizomes has been time-tested and documented during the long-period of traditional use. However, there is still a scope of research on the mechanism of action of several other aforementioned therapeutic activities. Moreover, among the 32 species, only nine species have been experimentally reported to possess the pharmacological properties. There is a scope for phytochemical analysis and clinical efficacy trials with the rest of the 23 species. To date, 152 compounds have been isolated and characterized from the genus *Bergenia*.

The studies done so far on Bergenias have focused on investigation and assessment of germplasm resources, functional credentials of extracts and isolation of bioactive components, but the reports on cytological and molecular researches and standardization of plant-extracted drugs for product-development are still fragmentary. *B. hissarica* and *B. tianquanensis* are extremely rare species with very few reports on their biological activities. Therefore, the conservation of the *Bergenia* species is of immense concern from a biodiversity, ethnobotanical, and pharmacological perspective. Although the research is progressing on *Bergenia* species, their robust tissue culture protocols are yet to be discovered, as the publications [97,169,170,171,172,173,174] on tissue culture and germplasm maintenance activities are fragmentary (Supplementary Table S2). The present study proposes a wide scope for multiple benefits of *Bergenia* in the field of floriculture, health foods, pharmaceuticals, cosmetics, and many other industrial and economic ventures. To conclude, *Bergenia* species have huge potential to act as a panacea to numerous health-related maladies, and therefore their conservation is necessary.

## Figures and Tables

**Table 1:** Bioactive compounds and medicinal properties of different *Bergenia* species.

*Bergenia* Species	Distribution	Medicinal Property	Part Used	Chemical Constituents (Structure Number)	Reference(s)
*Bergenia ciliata* (Haw.) Sternb. 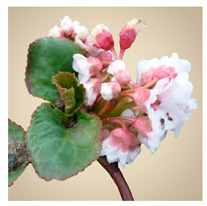	Central Asia, Afghanistan to China, Himalayan region. Altitude range (1800–3000 m)	Analgesic, Antiarrhythmic, Antiwrinkle, Antiasthma, Antibacterial, Anticancer, Antidiabetic, Antidiarrheal, Antidotary, Antiepileptic, Antiflatulent, Antifungal, Anti-haemorrhoidal, Antiviral, Anti-inflammatory, Antilithiatic, Antimalaria; Antimenorrhagic, Antiobesity, Antiophthalmia, Antioxidant, Antipyretic, Antispasmodic, Antiulcer, Burn wound healing, Deobstruent, Cerebroprotective, Diuretic, Ecbolic, Emmenagogue, Expectorant, Hepatoprotective, Immunomodulatory, Pulmonary affection	Whole plant	Bergenin **(1) ^a^** Catechin **(2) ^a^** Gallic acid **(3) ^a^** *β*-Sitosterol **(4) ^d^** Catechin-7-*O*-glucoside **(5) ^a^** Afzelechin **(6) ^a^** Quercetin-3-*O*-*β*-d-xylopyranoside **(7) ^a^** Quercetin-3-*O*-*α*-l-arbinofuranoxide **(8) ^a^** Eryodictiol-7-*O*-*β*-d-glucopyranoside **(9) ^a^** Arbutin **(10) ^c^** 6-*O*-*p*-Hydroxybenzoyl arbutin **(11) ^a^** 4-*O*-Galloylbergenin **(12) ^a^** 11-*O*-Galloylbergenin **(13) ^a^** *p*-Hydroxybenzoic acid **(14) ^f^** Protocatechuic acid **(15) ^a^** 6-*O*-Protocatechuoyl arbutin **(16) ^a^** 11-*O*-*p*-Hydroxybenzoyl bergenin **(17) ^a^** 11-*O*-Protocatechuoyl bergenin **(18) ^a^** 6-*O*-*p*-Hydroxybenzoyl parasorboside **(19) ^a^**	[11,16,31,43,72,73,74,75,76,77,78,79,80,81,82,83,84,85,86,87,88,89,90,91]
*Bergenia crassifolia* (L.) Fritsch [Synonym: *Bergenia cordifolia* (Haw.) Sternb.] 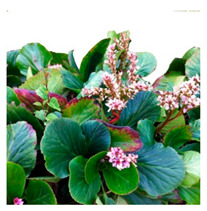	North Eastern Asia. Altitude range (200–2000 m)	Antihypertensive, Anti-inflammatory, Antilithiatic, Antiobesity, Antioxidant, Antipyretic, Cerebroprotective, Diuretic, Hepatoprotective, Immunomodulatory	Whole plant	Ellagitannins **(20) ^a^** Gallic acid **(3) ^a^** Arbutin **(10) ^c^** Bergenin **(1) ^a^** Caffeoylquinic acid **(21) ^c^** Monogalloylquinic acid **(22) ^c^** 2,4,6-Tri-*O*-galloyl-*β*-d-glucose **(23) ^a^** Pedunculagin **(24) ^a^** Tellimagrandin I **(25) ^a^** Catechin-7-*O*-*β*-d-glucoside **(5) ^a^** Paashanolactone **(26) ^b^** Catechin **(2) ^a^** *β*-Sitosterol **(4) ^b^** 2,4-Heptadienal **(27) ^f^** Benzaldehyde **(28) ^f^** Benzeneacetaldehyde **(29) ^f^** Decadienal **(30) ^f^** Decanal **(31) ^f^** Dimethylcyclohexene acetaldehyde **(32) ^f^** (*E*)-2-Decenal **(33) ^f^** (*E*)-2-Nonenal **(34) ^f^** Nonanal **(35) ^f^** *p*-Menthenal **(36) ^f^** (*E*)-*β*-Damascenone **(37) ^e^** (*E*)-*β*-Damascone **(38) ^e^** 3-Thujen-2-one **(39) ^e^** Caryophyllene **(40) ^e^** Cedranol **(41) ^e^** (*E*)-2-Decenol **(42) ^e^** Farnesol **(43) ^e^** Farnesyl acetone **(44) ^e^** Geraniol **(45) ^e^** Geranyl acetone **(46) ^e^** Hexahydrofarnesyl acetone **(47) ^e^** Ionone **(48) ^e^** Linalool **(49) ^e^** *m*-Mymene **(50) ^e^** Nerolidol **(51) ^e^** Phytol **(52) ^e^** *p*-Menth-1-en-4-ol **(53) ^e^** Prenol **(54) ^e^** Thymol **(55) ^e^** *α*-Bisabolol **(56) ^e^** *α*-Bisabololoxide B **(57) ^e^** *α*-Cadinol **(58) ^e^** *α*-Terpineol **(59) ^e^** *β*-Elemene **(60) ^e^** *β*-Eudesmol **(61) ^e^** *δ*-Cadinene **(62) ^e^** 11-*O*-(*p*-Hydroxybezoyl)bergenin **(63) ^a^** 3,11-Di-*O*-galloylbergenin **(64) ^a^** 4,11-Di-*O*-galloylbergenin **(65) ^a^** Bergapten **(66) ^a^** Kaempferol-3-*O*-xylosylgalactoside **(67) ^a^** Kaempferol-3-*O*-xylosylglucoside **(68) ^a^** Kaempferol-3-*O*-arabinoside **(69) ^a^** Kaempferol-3-*O*-rutinoside **(70) ^a^** Norathyriol **(71) ^a^** Norbergenin **(72) ^a^** Quercetin-3-*O*-xylosylgalactoside **(73) ^a^** Quercetin-3-*O*-xylosylglucoside **(74) ^a^** Quercetin-3-*O*-arabinoside **(75) ^a^** Quercetin-3-*O*-galactoside **(76) ^a^** Quercetin-3-*O*-glucoside **(77) ^a^** Quercetin-3-*O*-rhamnoside **(78) ^a^** Quercetin-3-*O*-rutinoside **(79) ^a^** Quercetin-3-*O*-xyloside **(80) ^a^** Trihydroxycoumarin **(81) ^a^** (+)-Catechin-3,5-di-*O*-gallate **(82) ^a^** (+)-Catechin-3-*O*-gallate **(83) ^a^** 1,2,4,6-Tetra-*O*-galloy-*β*-d-glucopyranose **(84) ^a^** 1-*O*-Galloylglucose **(85) ^a^** 2-*O*-Caffeoylarbutin **(86) ^a^** 6-*O*-Galloylarbutin **(87) ^a^** Ellagic acid **(88) ^a^** Hydroquinone **(89) ^a^** *p*-Galloyloxyphenyl-*β*-d-glucoside **(90) ^a^** Pyrogallol **(91) ^a^** Acetylsalicylic acid **(92) ^f^** Fumaric acid **(93) ^f^** Furancarboxylic acid **(94) ^f^** Protocatechuic acid **(15) ^f^** Malic acid **(95) ^f^** Quinic acid **(96) ^f^** 4-Methoxystyrene **(97) ^f^** 9,12-Octadecadienoic acid **(98) ^f^** 9-Octadecenoic acid **(99) ^f^** Decanoic acid **(100) ^f^** Dodecanoic acid **(101) ^f^** Hexadecanoic acid **(102) ^f^** *n*-Cetyl alcohol **(103) ^f^** *n*-Eicosanol **(104) ^f^** *n*-Hentriacontane **(105) ^f^** *n*-Heptacosane **(106) ^f^** *n*-Nonacosane **(107) ^f^** Nonanoic acid **(108) ^f^** *n*-pentacosane **(109) ^f^** Pentadecanoic acid **(110) ^f^** Rhododendrin **(111) ^f^** Stearic acid **(112) ^f^** Tetradecanoic acid **(113) ^f^** Tetramethyl hexadecenol **(114) ^f^** Trimethyl dihydronaphthalene **(115) ^f^** Trimethyl-3-methylene hexadecatetraene **(116) ^f^**	[14,28,31,64,79,85,92,93,94,95,96,97,98,99,100,101,102,103,104]
*Bergenia emeiensis* C.Y. Wu ex J.T. Pan. 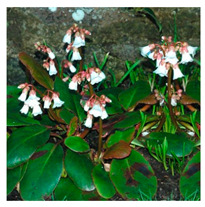	China. Altitude range (1600–4200 m)	Antiwrinkle, Anti-inflammatory, Antiobesity, Antioxidant	Whole plant	Bergenin **(1) ^a^** Tannic acid **(117) ^a^** Arbutin **(10) ^c^**	[31,65,85,105]
*Bergenia ligulata* Wall. Engl. [Accepted name: *Bergenia pacumbis* (Buch.-Ham. Ex D. Don.) C.Y. Wu & J.T. Pan] 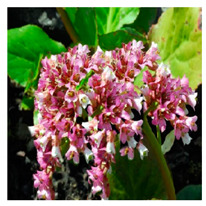	Temperate Himalayas. Altitude range (2134–3048 m)	Analgesic, Antiarrhythmic, Anticancer, Antidiabetic, Antifungal, Anti-haemorrhoidal, Antiviral, Anti-inflammatory, Antilithiatic, Antiprotozoal, Antipyretic, Antiscorbutic, Antispasmodic, Antitumor, Antiulcer, Astringent, burn wound healing, Cerebroprotective, Diuretic, Expectorant, Hepatoprotective, Immunomodulatory	Root, Rhizome	Bergenin **(1) ^a^** Gallic acid **(3) ^a^** Tannic acid **(117) ^a^** Arbutin **(10) ^c^** Catechin **(2) ^a^** *β*-Sitosterol **(4) ^b^** Stigmasterol **(118) ^d^** Afzelechin **(6) ^a^** 1,8-Cineole **(119) ^e^** Isovaleric acid **(120) ^f^** (+)-(6*S*)-Parasorbic acid **(121) ^b^** Terpinen-4-ol **(122) ^e^** (*Z*)-asarone **(123) ^f^** Leucocyanidin **(124) ^a^** Methyl gallate **(125) ^a^** Sitoinoside I **(126) ^d^** *β*-Sitosterol-d-glucoside **(127) ^d^** Avicularin **(128) ^a^** Eriodictyol-7-*O*-*β*-d-glucopyranoside **(9) ^a^** Reynoutrin **(129) ^a^** 11-*O*-Galloylbergenin **(13) ^a^** Pashaanolactone **(26) ^b^** Catechin-7-*O*-glucoside **(5) ^a^** Coumarin **(130) ^b^** 11-*O*-*p*-Hydroxybenzoyl bergenin **(17) ^a^** 11-*O*-Protocatechuoyl bergenin **(18) ^a^** 4-*O*-Galloylbergenin **(12) ^a^** 6-*O*-*p*-Hydroxybenzoyl arbutin **(11) ^a^** Hexan-5-olide **(131) ^b^** Quercetin **(132) ^a^** *β*-Sitosterol-d-glucoside **(127) ^d^**	[3,16,23,24,26,37,61,73,78,83,85,86,87,88,90,95,106,107,108,109,110,111,112,113,114,115,116,117,118,119,120,121,122,123,124,125,126,127]
*Bergenia purpurascens* (Hook.f. & Thomson) Engl. 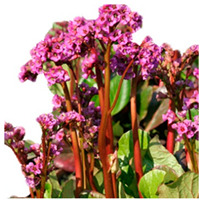	Eastern Himalayas. Altitude range (2800–4800 m)	Antibacterial, Anti-inflammatory, Antilithiatic, Antipyretic	Rhizome	Catechin **(2) ^a^** Gallic acid **(3) ^a^** Bergenin **(1) ^a^** Arbutin **(10) ^c^** 1,2,3,4,6-Penta-*O*-galloyl-*β*-d-glucose **(133) ^a^** 4,6-Di-*O*-galloyl-*β*-d-glucose **(134) ^a^** 6-*O*-Galloylarbutin **(87) ^a^** 11-*O*-Galloylbergenin **(13) ^a^** 4-*O*-Galloylbergenin **(12) ^a^** 2,3,4,6-Tetra-*O*-galloyl-*β*-d-glucose **(84) ^a^** Procyanidin B1 **(135) ^a^** 2,4,6-Tri-*O*-galloyl-*β*-d-glucose **(23) ^a^** Procyanidin B3 **(136) ^a^**	[31,44,56,62,85,95,100,128,129,130,131,132,133]
*Bergenia scopulosa* (T.P. Wang) 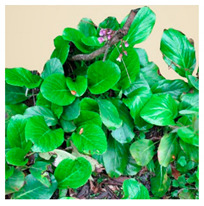	China. Altitude range (2400–3600 m)	Anti-hypertensive, Anti-inflammatory, Antiobesity, Antioxidant, Antitussive, Cerebroprotective, Diuretic, Hepatoprotective, Immunomodulatory	Leaf, Root, Rhizome	Bergenin **(1) ^a^** Arbutin **(10) ^c^** Catechin **(2) ^a^** *β*-Sitosterol **(4) ^d^** 6-*O*-Galloylarbutin **(87) ^a^** Catechin-7-*O*-*β*-d-glucopyranoside **(5) ^a^** Phenylalanine **(137) ^f^** Succinic acid **(138) ^f^** Protocatechuic acid **(15) ^a^** Gallic acid **(3) ^a^** Methyl gallate **(125) ^a^** Quercetin **(133) ^a^** Hyperoside **(139) ^a^** Quercetin-3-*O*-rutinoside **(79) ^a^** Afzelin **(140) ^a^** Chrysophanol-8-*O*-*β*-d-glucopyranoside **(141) ^c^** 11-*O*-Galloylbergenin **(13) ^a^**	[31,59,66,67,85,134,135,136,137,138]
*Bergenia stracheyi* (Hook. f. & Thomas) Engl. 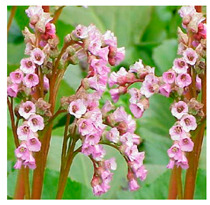	Afghanistan, Pakistan, Nepal. Altitude range (3000–4600 m)	Antifungal, Anti-haemorrhoidal, Anti-inflammatory, Antilithiatic, Antiobesity, Antioxidant, Poultice to treat the stiff joints	Rhizome	Bergenin **(1) ^a^** (+)-Catechin-3-*O*-gallate **(83) ^a^** Gallic acid **(3) ^a^** Tannic acid **(117) ^a^** Phytol **(142) ^e^** Caryophyllene **(40) ^e^** Damascenone **(143) ^f^** *β*-Eudesmol **(144) ^e^** 3-Methyl-2-buten-1-ol **(145) ^e^**	[13,83,85,86,88,90,116,139,140,141]
*Bergenia hissarica* (A. Boriss.) 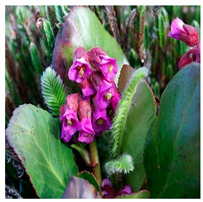	Central Asia, Uzbekistan, Hissar. Altitude range (1200–1600 m)	Stimulantlaxative, Neuroprotective, Antioxidant	Root, Rhizome	Aloe emodin **(146) ^c^** Aloeemodin-8-*O*-*β*-d-glucoside **(147) ^c^** Chrysophanein **(148) ^c^** Emodin-1-*O*-*β*-d-glucoside **(149) ^c^** Physeion **(150) ^d^**	[142,143]
*Bergenia tianquanesis* (J.T. Pan) 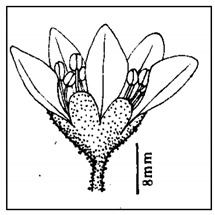	China. Altitude range (2200–3400 m)	Not reported			[29,32]

^a^ Polyphenols; ^b^ Lactones; ^c^ Quinones; ^d^ Sterols; ^e^ Terpenes; ^f^ Others. Number beside each bioactive compound represents the structure number as shown in Supplementary Figure S1.

## Supplementary Materials

The following are available online: Figure S1: Chemical structures of isolated and characterized phytochemicals from *Bergenia* species, Table S1: Botanical description of *Bergenia* species, Table S2: Tissue culture reports of *Bergenia* species.
