# Supplementary material for: Bergenia Genus: Traditional Uses, Phytochemistry and Pharmacology
Source: Molecules. 2020 Nov 26;25(23):5555. doi: 10.3390/molecules25235555 (PMC7730924; doi:10.3390/molecules25235555)
Supplement: Supplementary file 1 [file molecules-25-05555-s001.pdf]

Bergenia Genus: Traditional Uses, Phytochemistry and Pharmacology

Bhupendra Koul [1,*], Arvind Kumar [2], Dhananjay Yadav [3,*] and Jun-O Jin [3,4,*]

1. School of Bioengineering and Biosciences, Lovely Professional University, Phagwara 144411, Punjab, India
2. Research Center for Chromatography and Mass Spectrometry, CROM-MASS, CIBIMOL-CENIVAM, Industrial University of Santander, Carrera 27, Calle 9, Edificio 45, Bucaramanga 680002, Colombia; arvindtomer81@gmail.com
3. Department of Medical Biotechnology, Yeungnam University, Gyeongsan 38541, Korea
4. Research Institute of Cell Culture, Yeungnam University, Gyeongsan 38541, Korea

* Correspondence: bhupendra.18673@lpu.co.in (B.K.); dhanyadav16481@gmail.com (D.Y.); jinjo@yu.ac.kr (J.-O.J.); Tel.: +91-9454320518 (B.K.); +82-1022021191(D.Y.); +82-53-810-3033 (J.-O.J.); Fax: +82-53-810-4769 (J.-O.J.)

Supplementary Fig. 1. Chemical structures of isolated and characterized phytochemicals from *Bergenia* species.

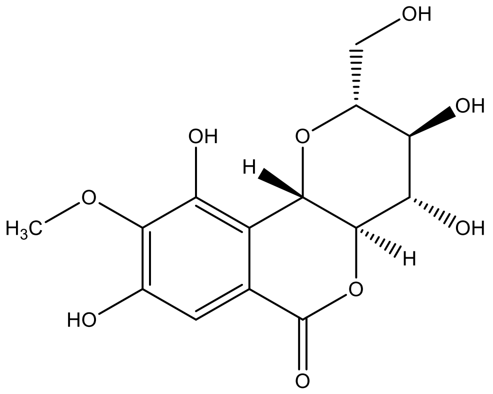

(1)

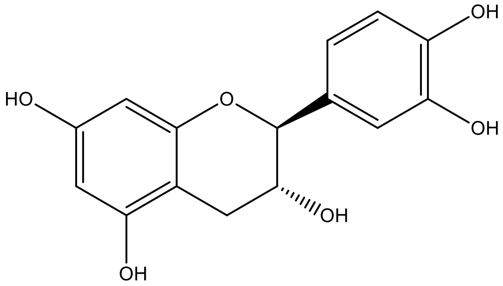

(2)

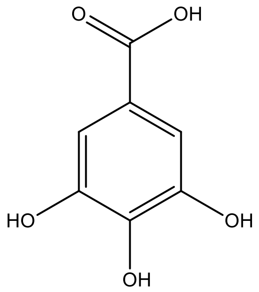

(3)

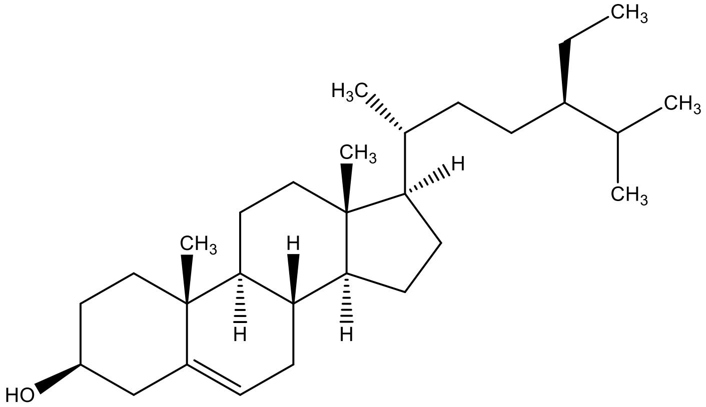

(4)

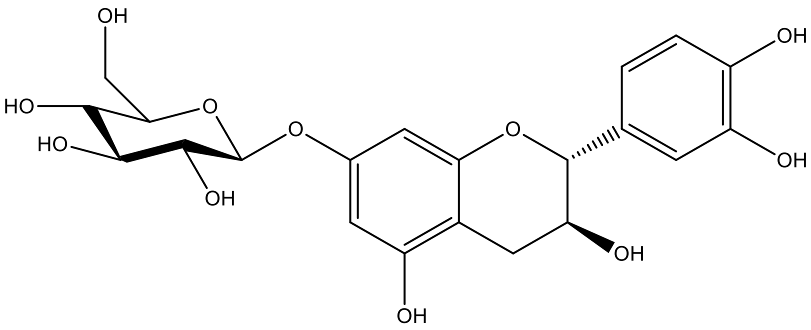

(5)

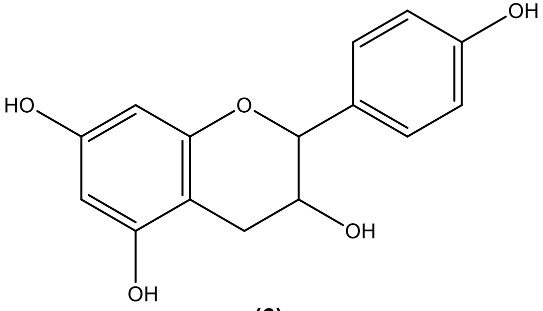
OH
HO
O
OH
OH

(6)

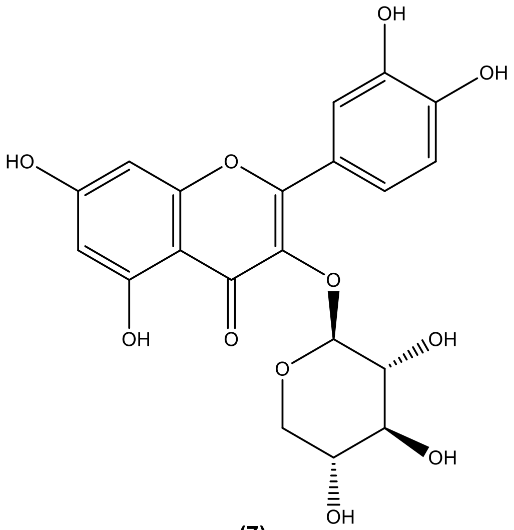
OH
OH
HO
O
O
OH
O
O
OH
OH
OH
OH

(7)

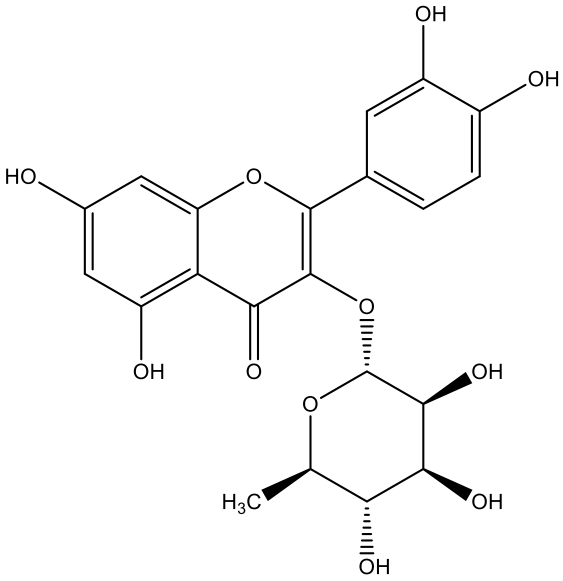
OH
OH
HO
O
O
OH
O
O
OH
H_3C
OH
OH

(8)

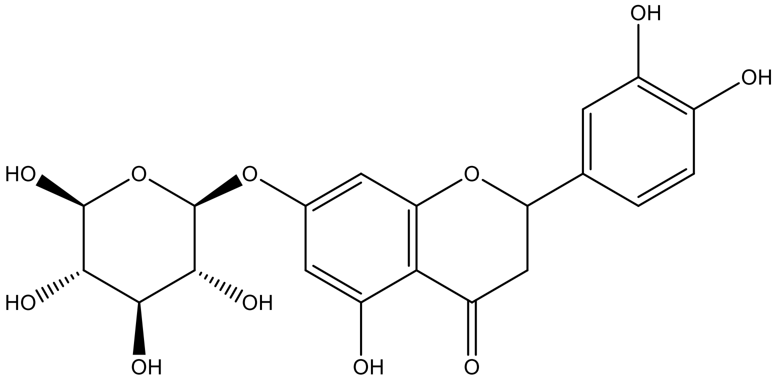
OH
OH
HO
O
O
O
HO
OH
OH
OH
OH
O

(9)

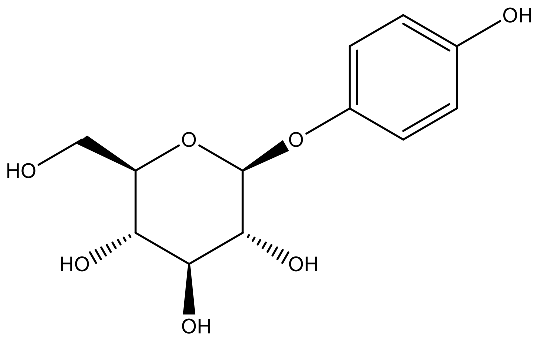
OH
HO
O
O
HO
OH
OH

(10)

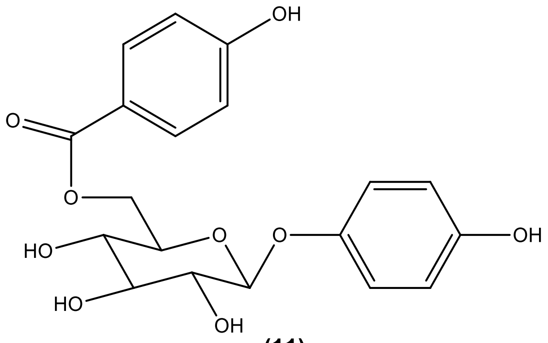
OH
O
O
HO
O
O
OH
HO
OH

(11)

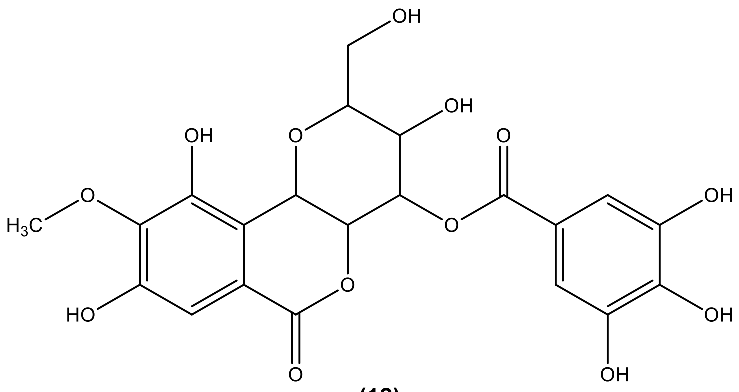
OH
OH
OH
O
O
H3C
O
O
OH
O
OH
HO
O
OH
OH

(12)

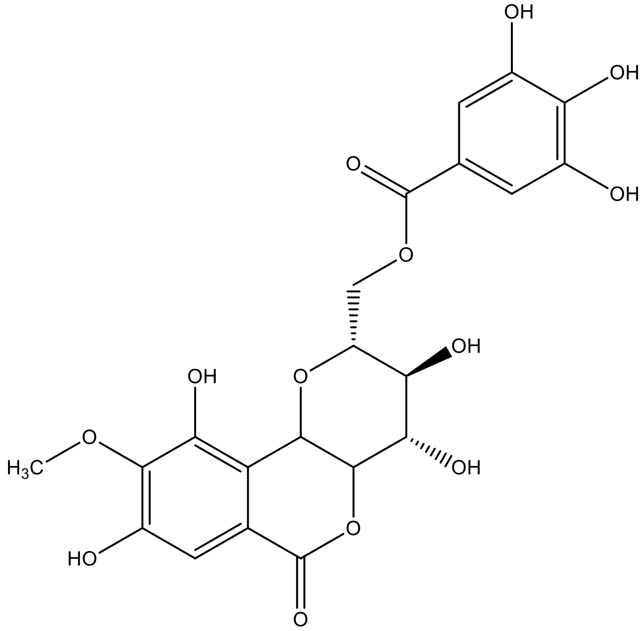
OH
OH
O
OH
O
OH
O
OH
H3C
O
OH
HO
O
O

(13)

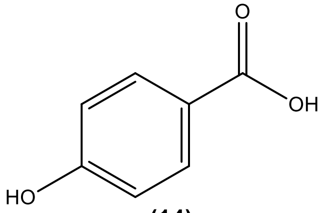
O
OH
HO

(14)

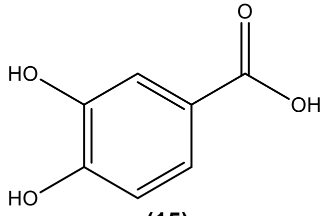
O
HO
OH
HO

(15)

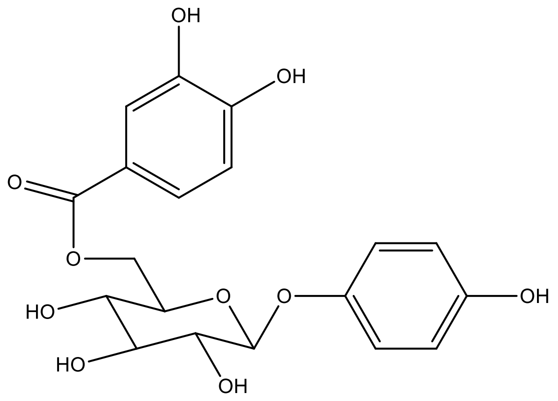

(16)

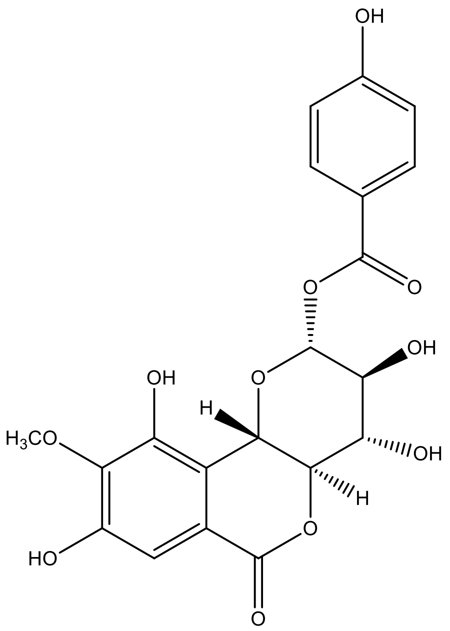

(17)

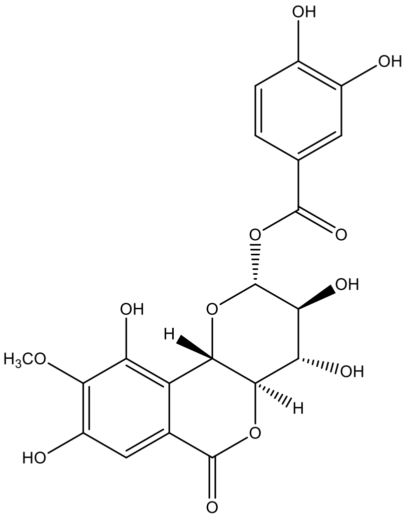

(18)

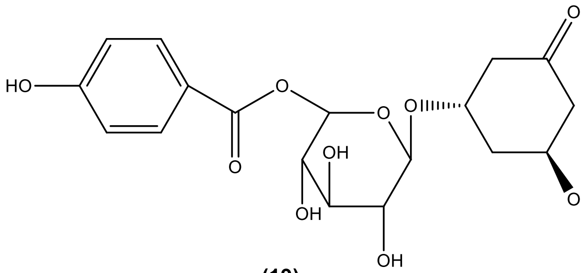

(19)

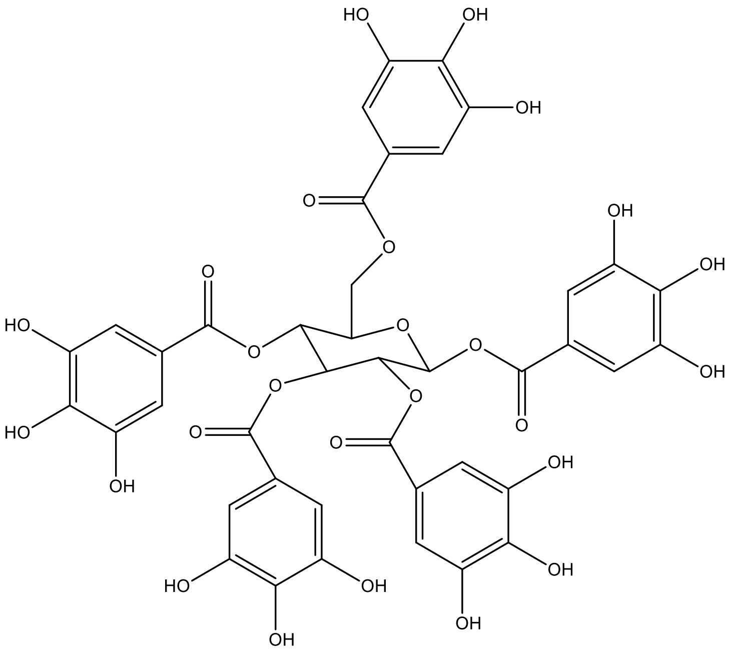

(20)

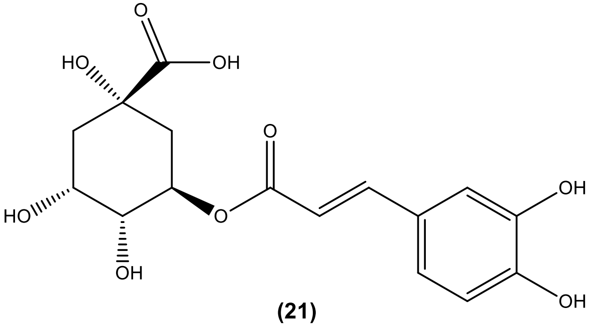

(21)

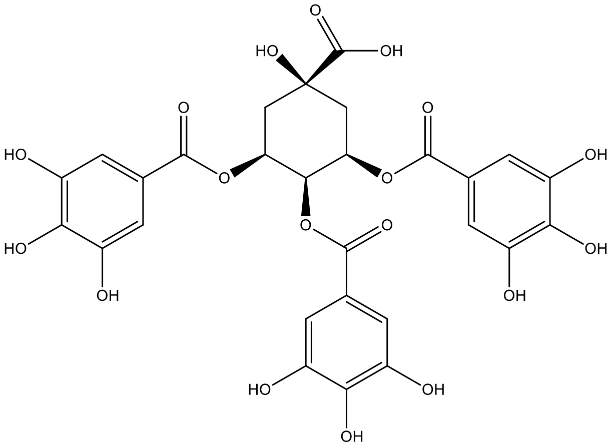

(22)

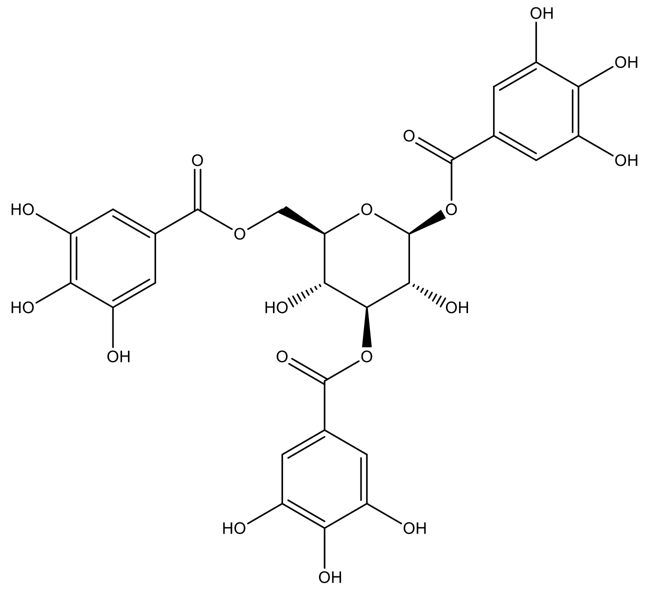

(23)

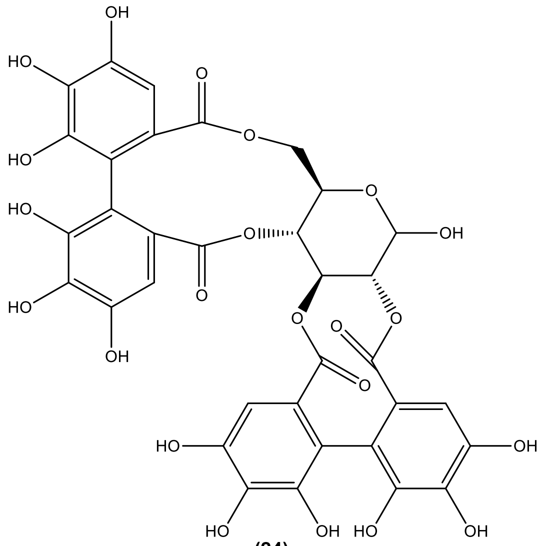

(24)

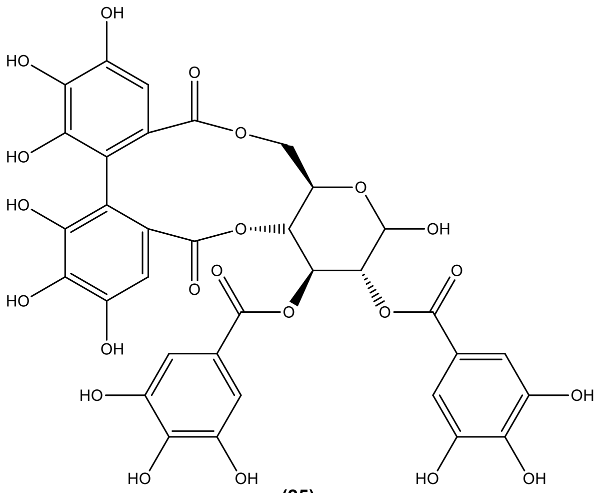

(25)

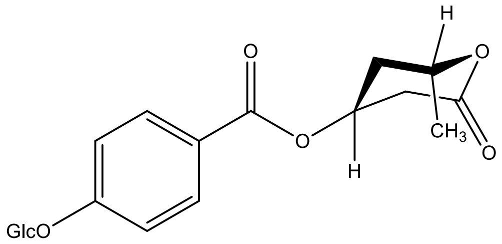

(26)

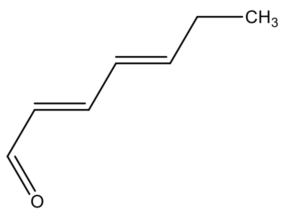

(27)

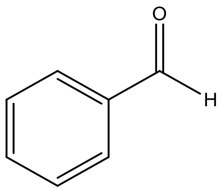

(28)

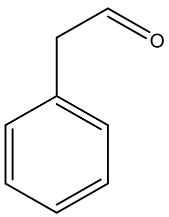

(29)

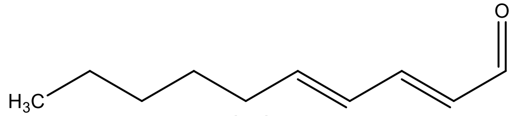

(30)

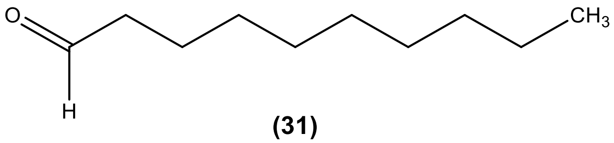

(31)

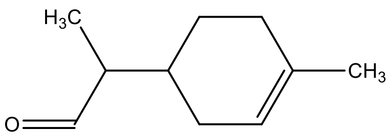

(32)

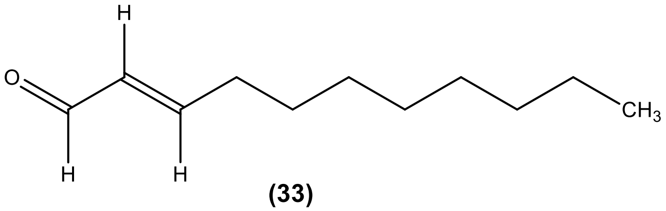

(33)

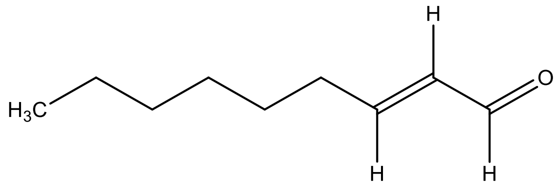

(34)

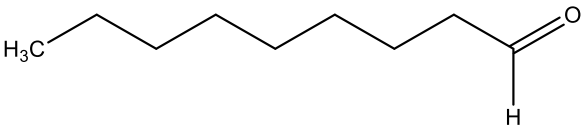

(35)

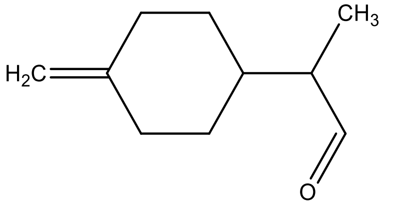

(36)

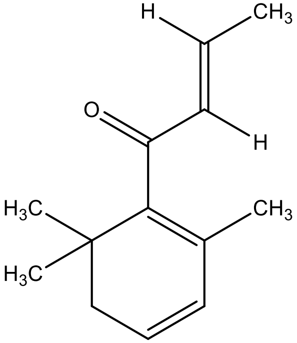

(37)

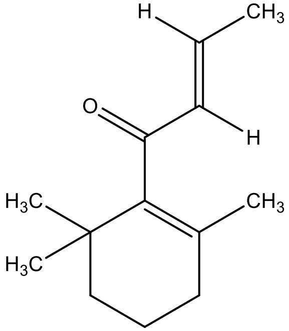

(38)

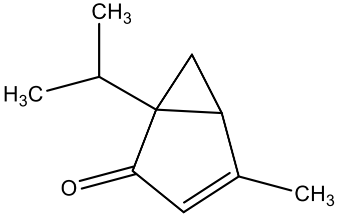

(39)

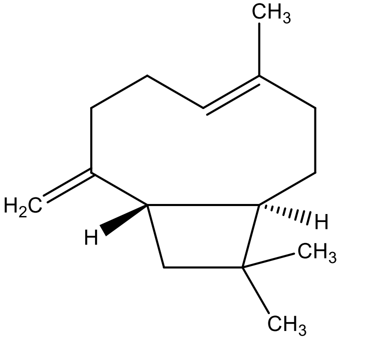

(40)

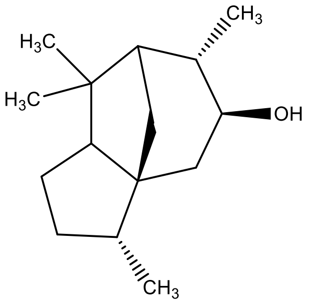

(41)

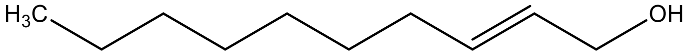

(42)

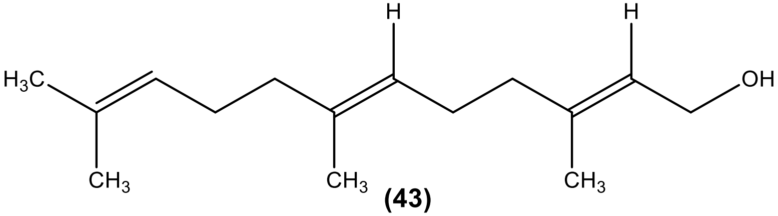

(43)

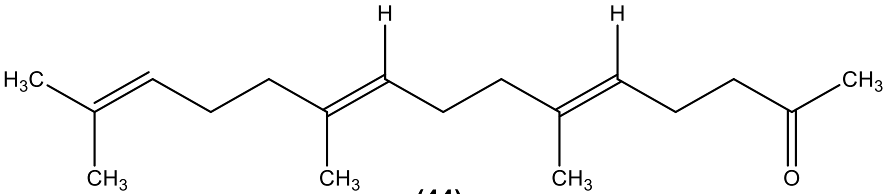

(44)

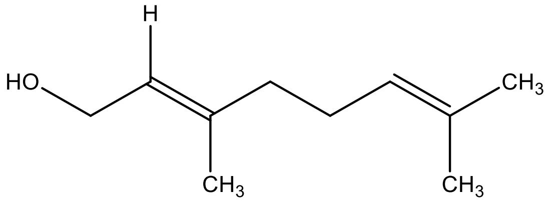

(45)

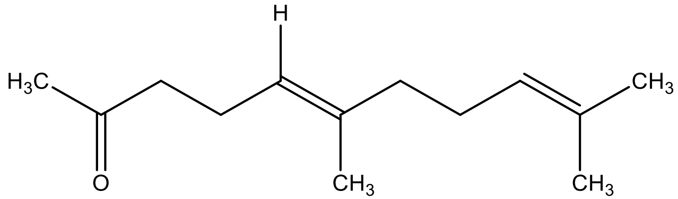

(46)

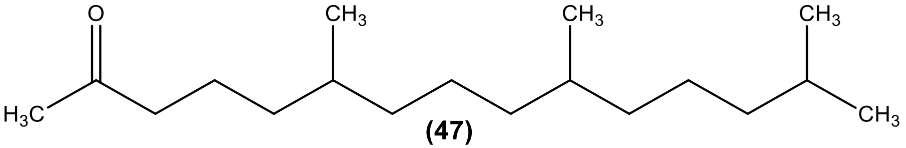

(47)

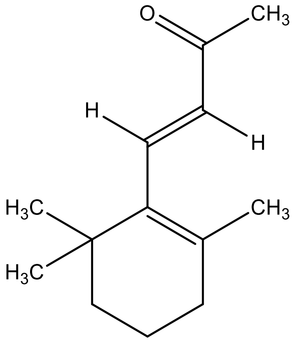

(48)

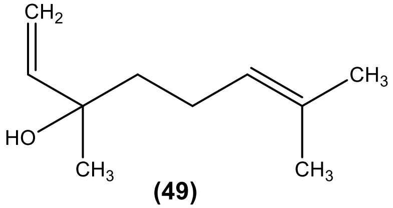

(49)

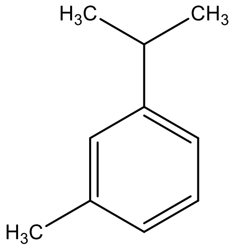

(50)

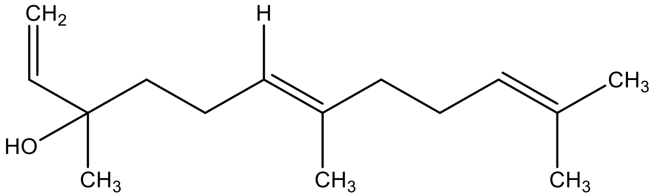

(51)

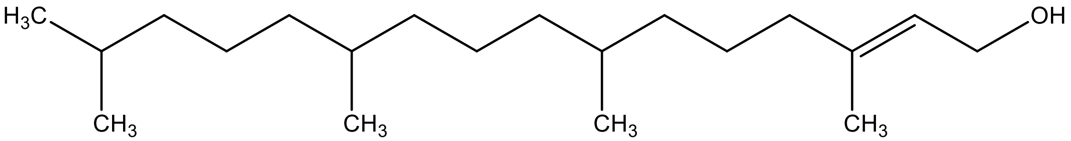

(52)

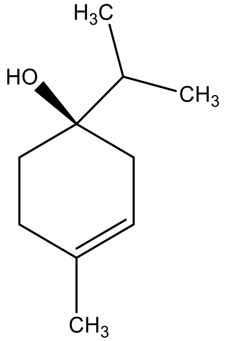

(53)

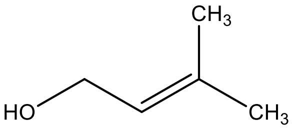

(54)

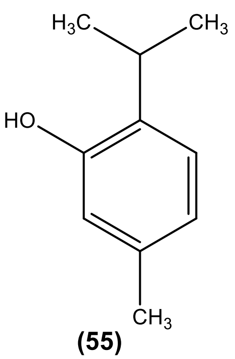

(55)

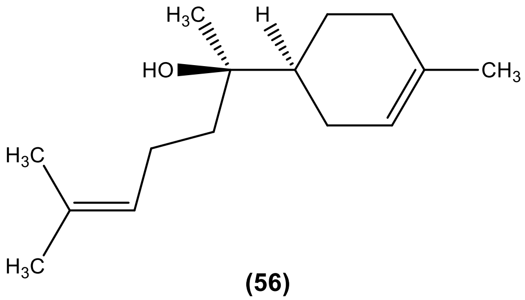

(56)

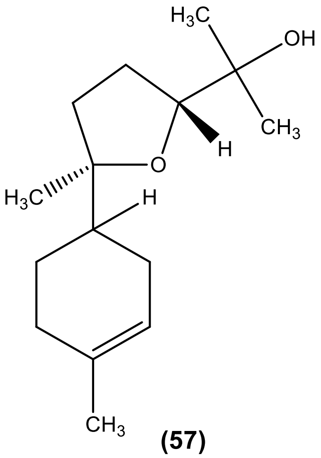

(57)

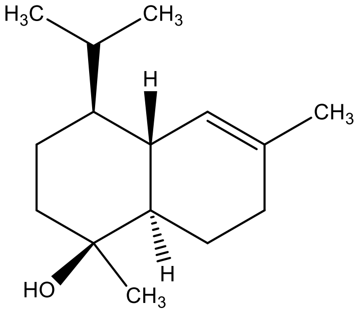

(58)

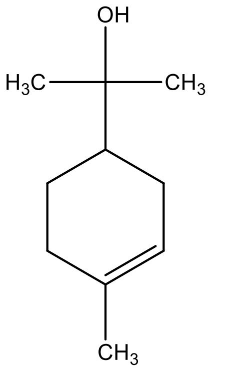

(59)

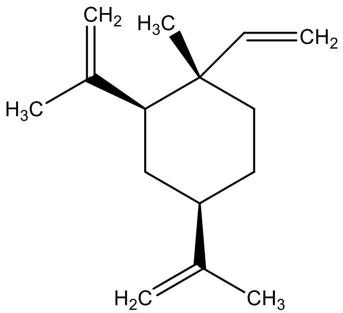

(60)

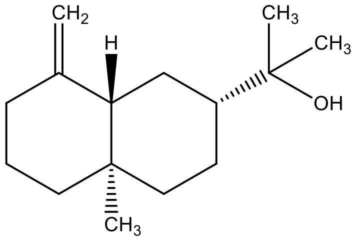

(61)

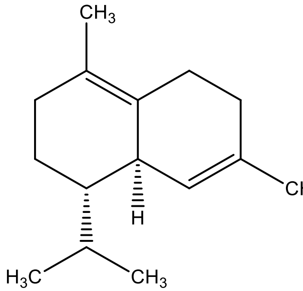

(62)

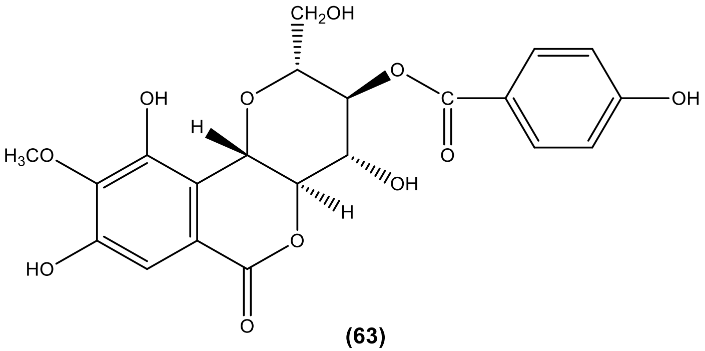

(63)

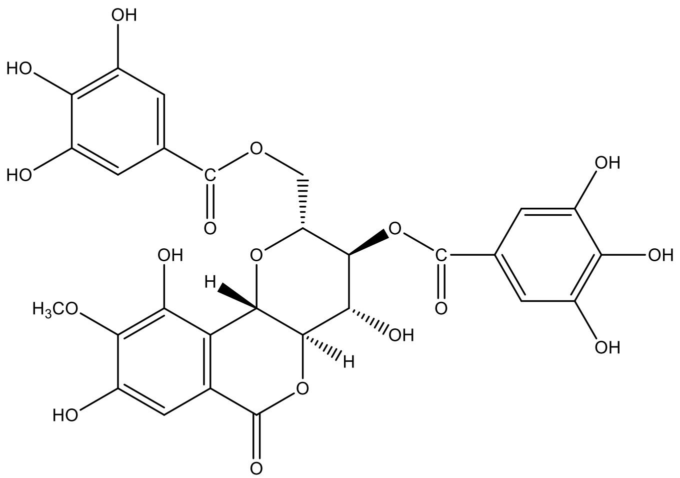

(64)

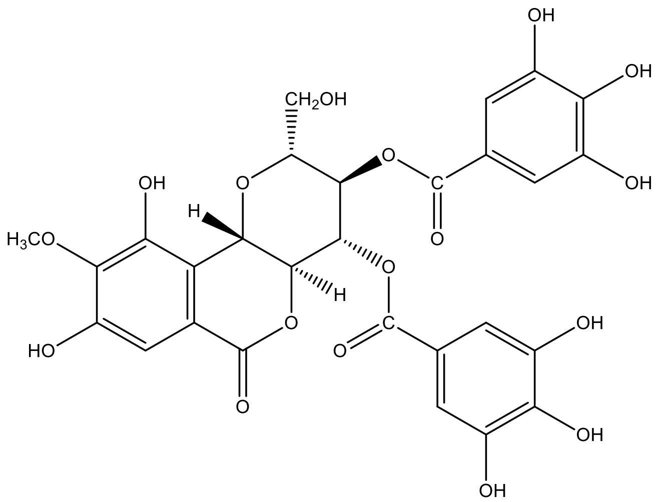

(65)

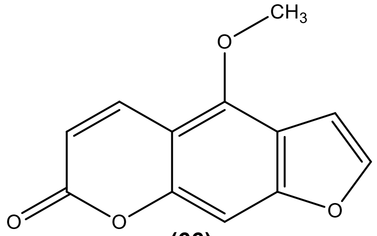

(66)

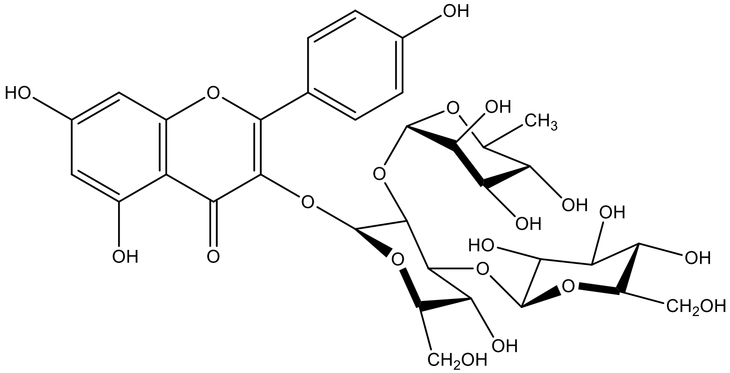

(67)

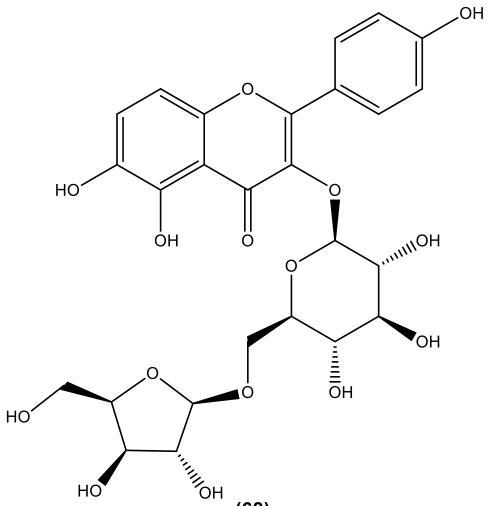

(68)

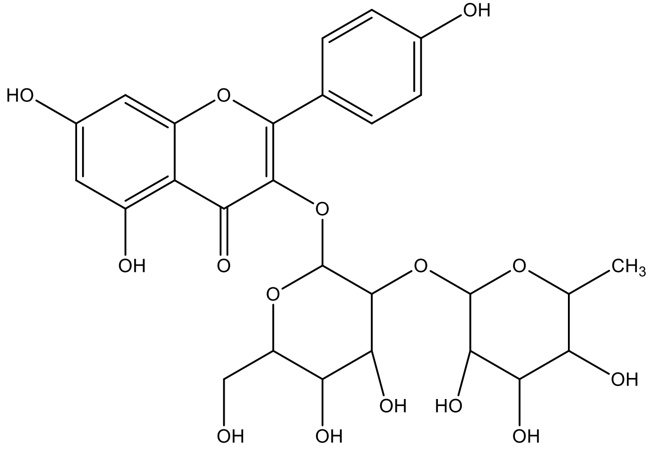
OH
HO
O
O
OH
O
O
O
CH3
O
OH
OH
OH
HO
OH
OH
OH

(70)

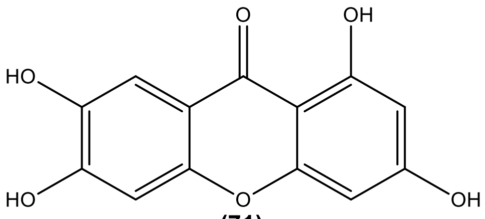
O
OH
HO
HO
O
OH

(71)

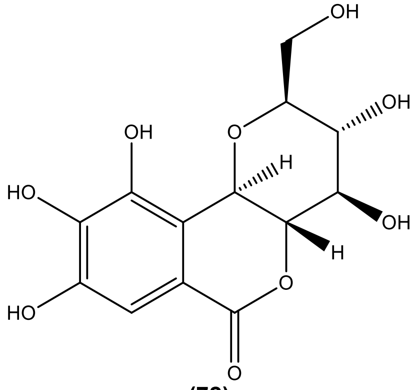
OH
OH
OH
OH
HO
O
H
HO
O
H
HO
O

(72)

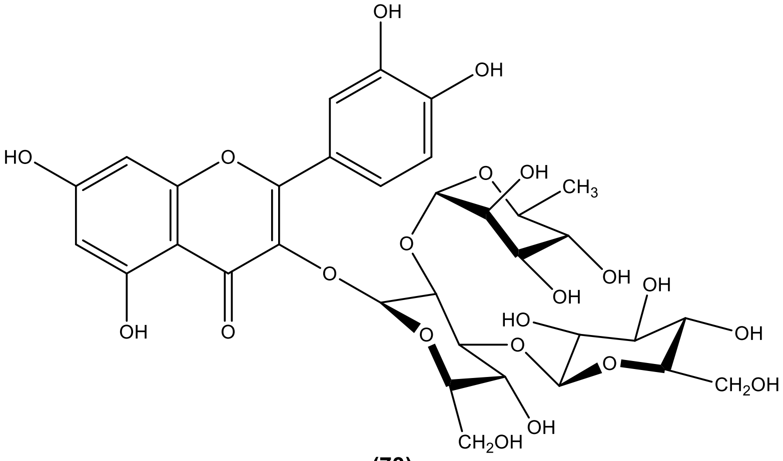
OH
OH
HO
O
O
CH3
OH
O
O
OH
HO
OH
OH
OH
HO
O
O
CH2OH
OH
CH2OH

(73)

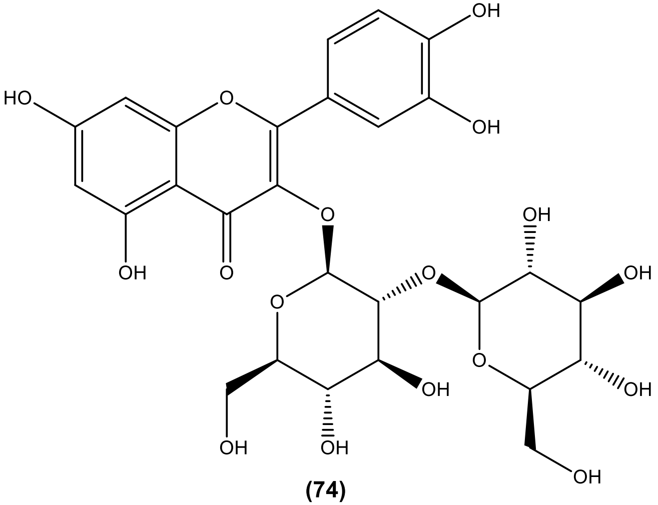
OH
HO
O
OH
O
OH
O
OH
O
OH
O
OH
O
O
OH
OH
OH
OH
OH

(74)

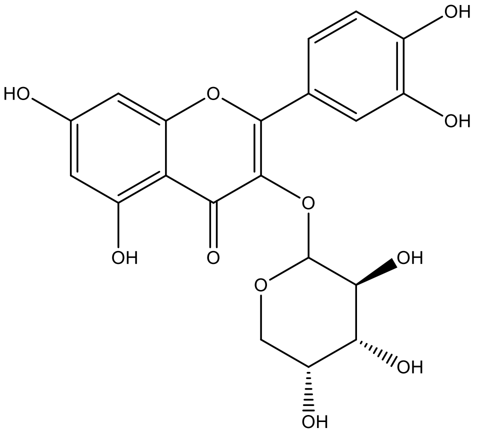

(75)

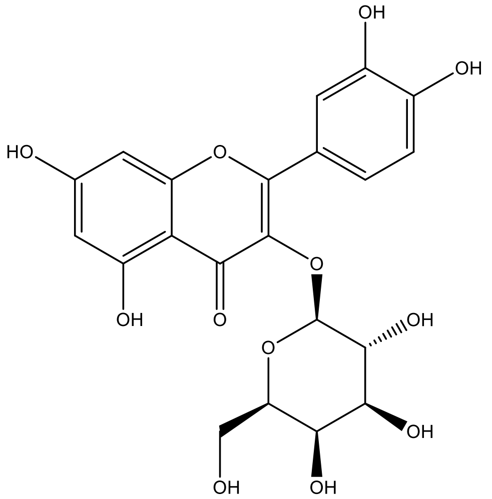

(76)

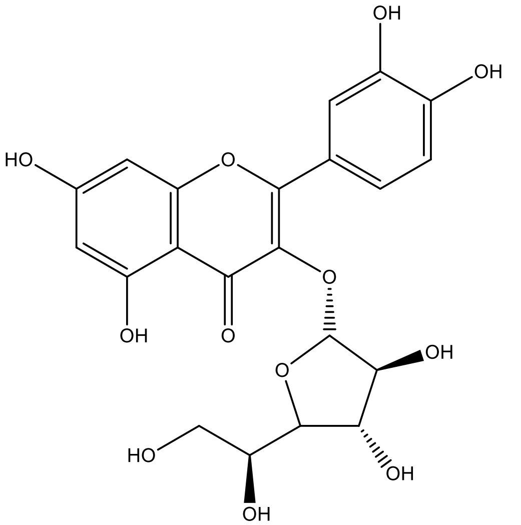

(77)

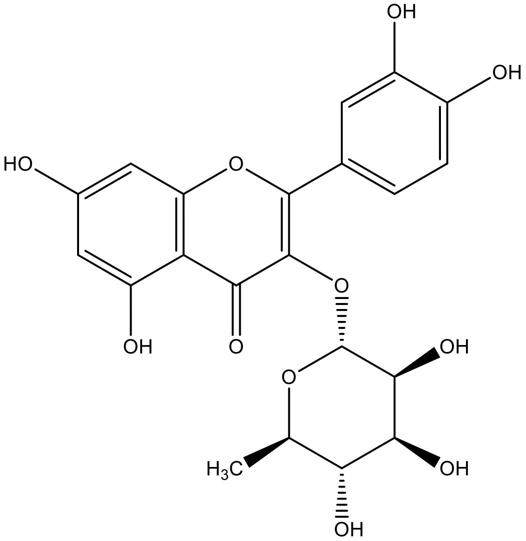

(78)

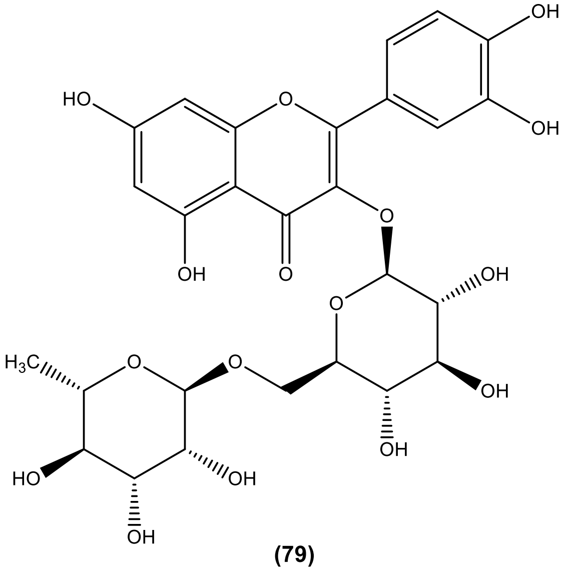

(79)

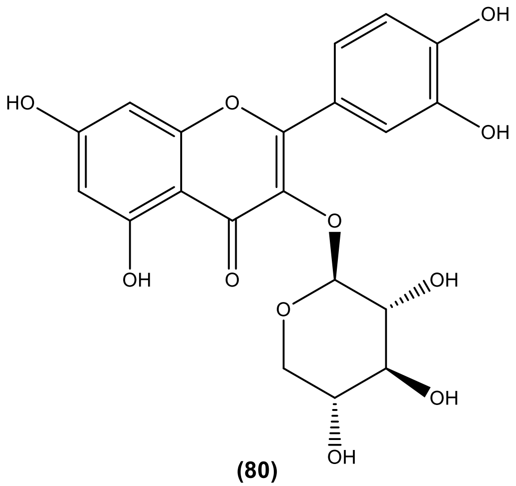

(80)

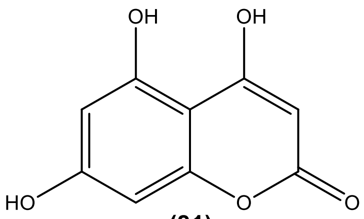

(81)

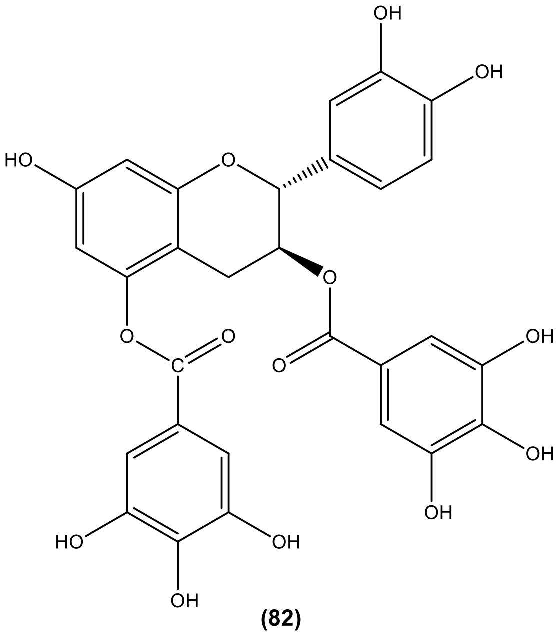

(82)

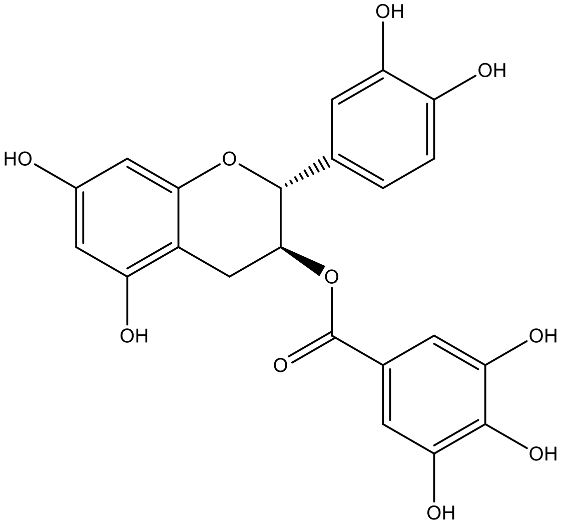

(83)

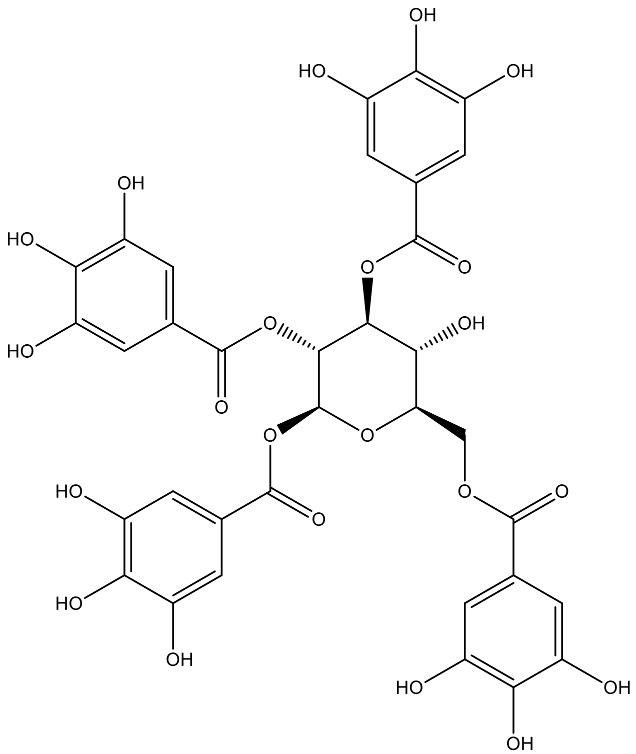

(84)

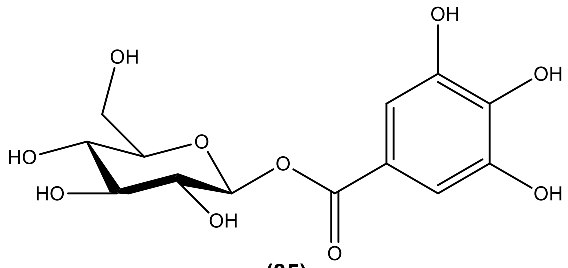

(85)

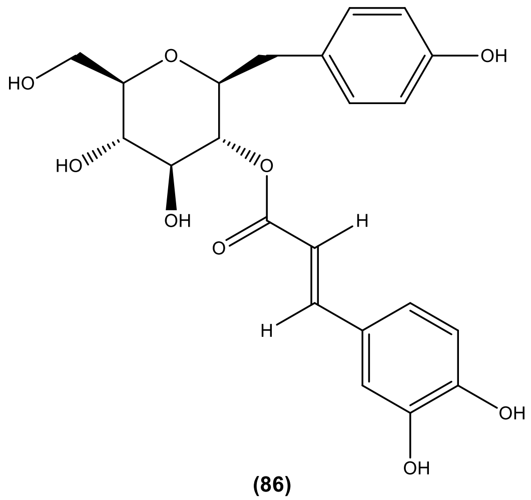

(86)

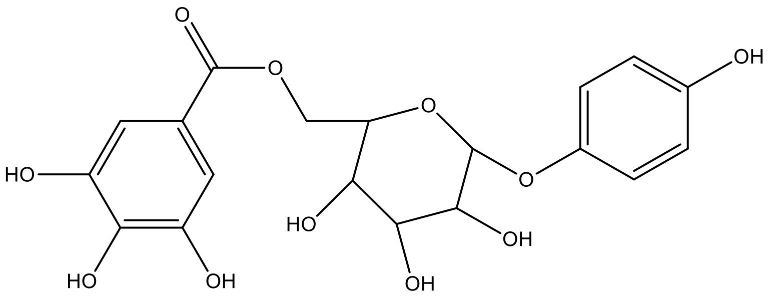

(87)

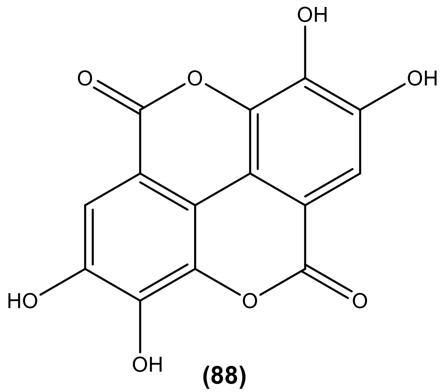

(88)

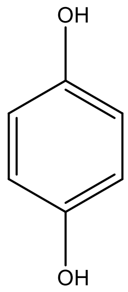

(89)

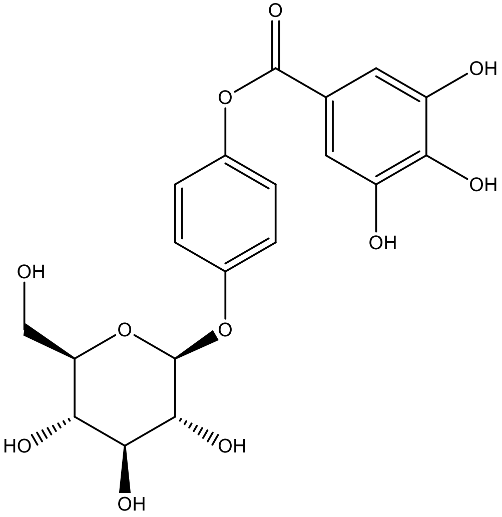

(90)

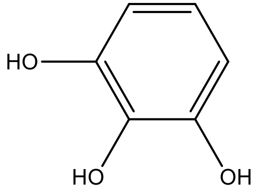

(91)

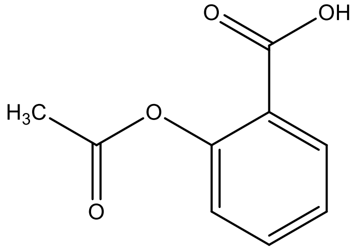

(92)

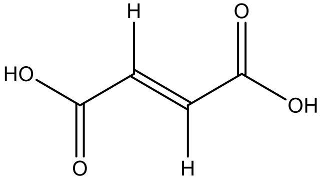

(93)

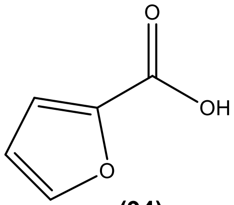

(94)

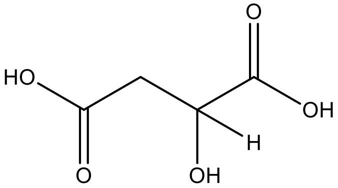

(95)

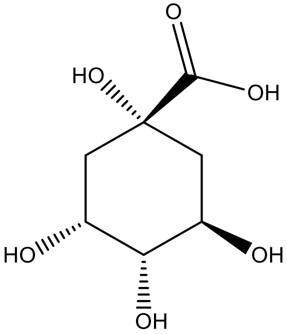

(96)

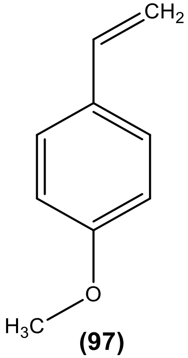

(97)

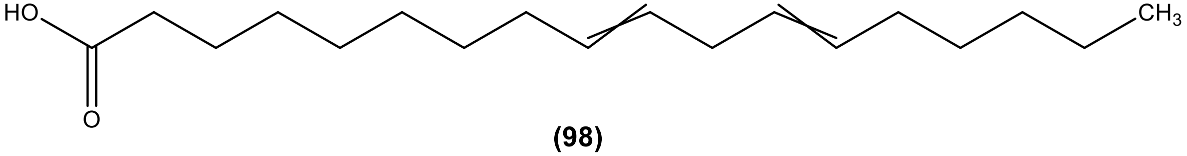

(98)

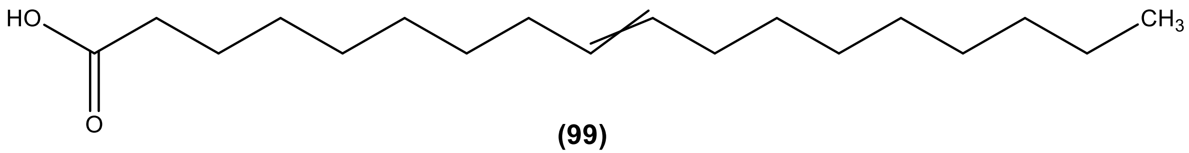

(99)

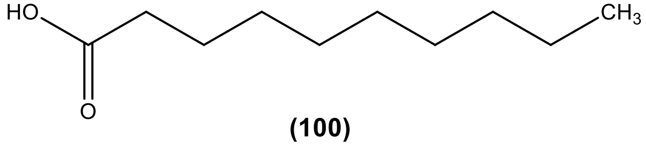

(100)

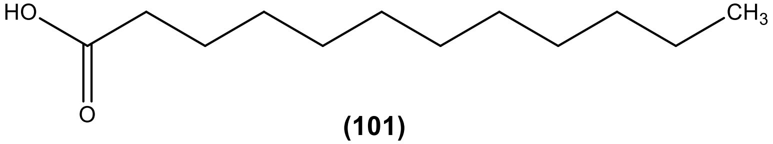

(101)

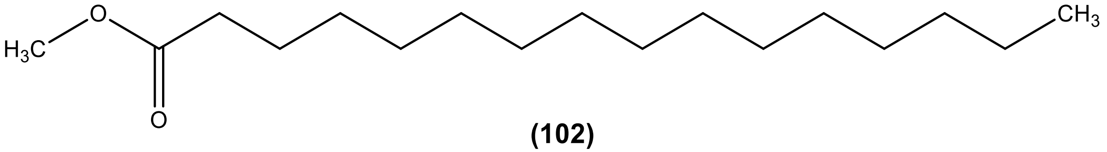

(102)

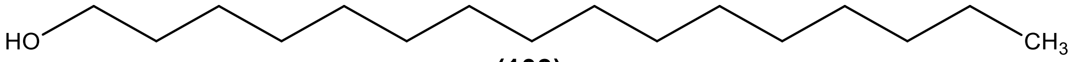

(103)

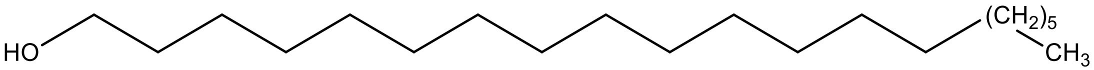

(104)

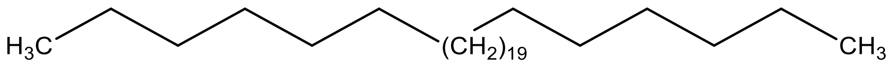

(105)

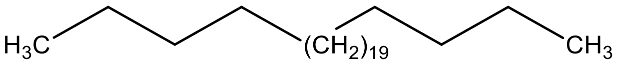

(106)

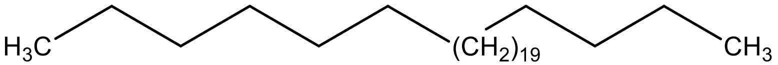

(107)

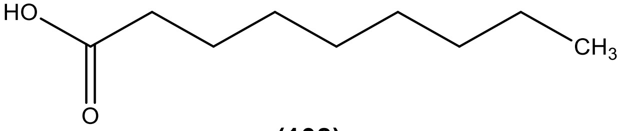

(108)

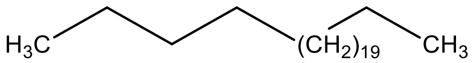

(109)

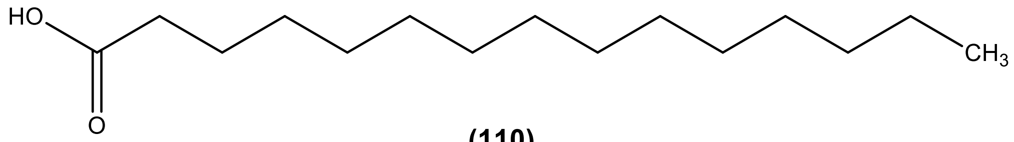

(110)

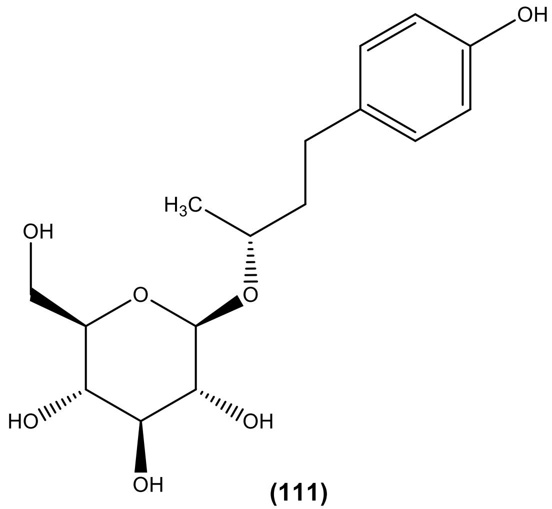

(111)

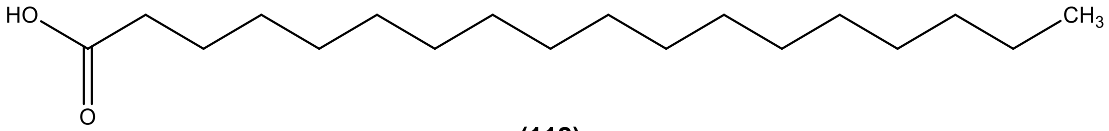

(112)

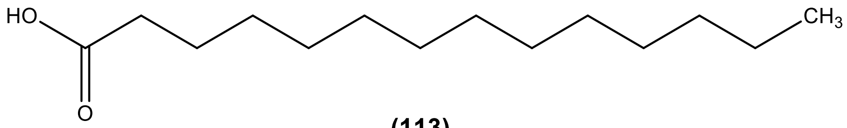

(113)

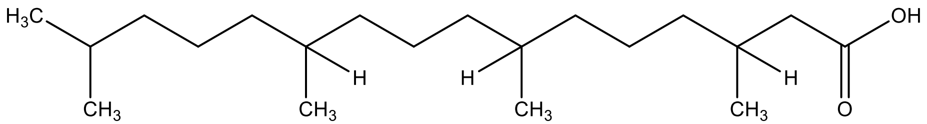

(114)

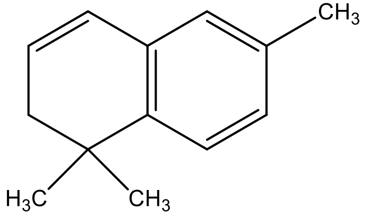

(115)

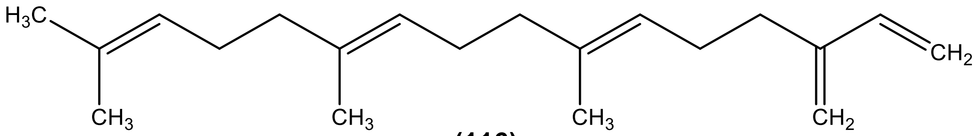

(116)

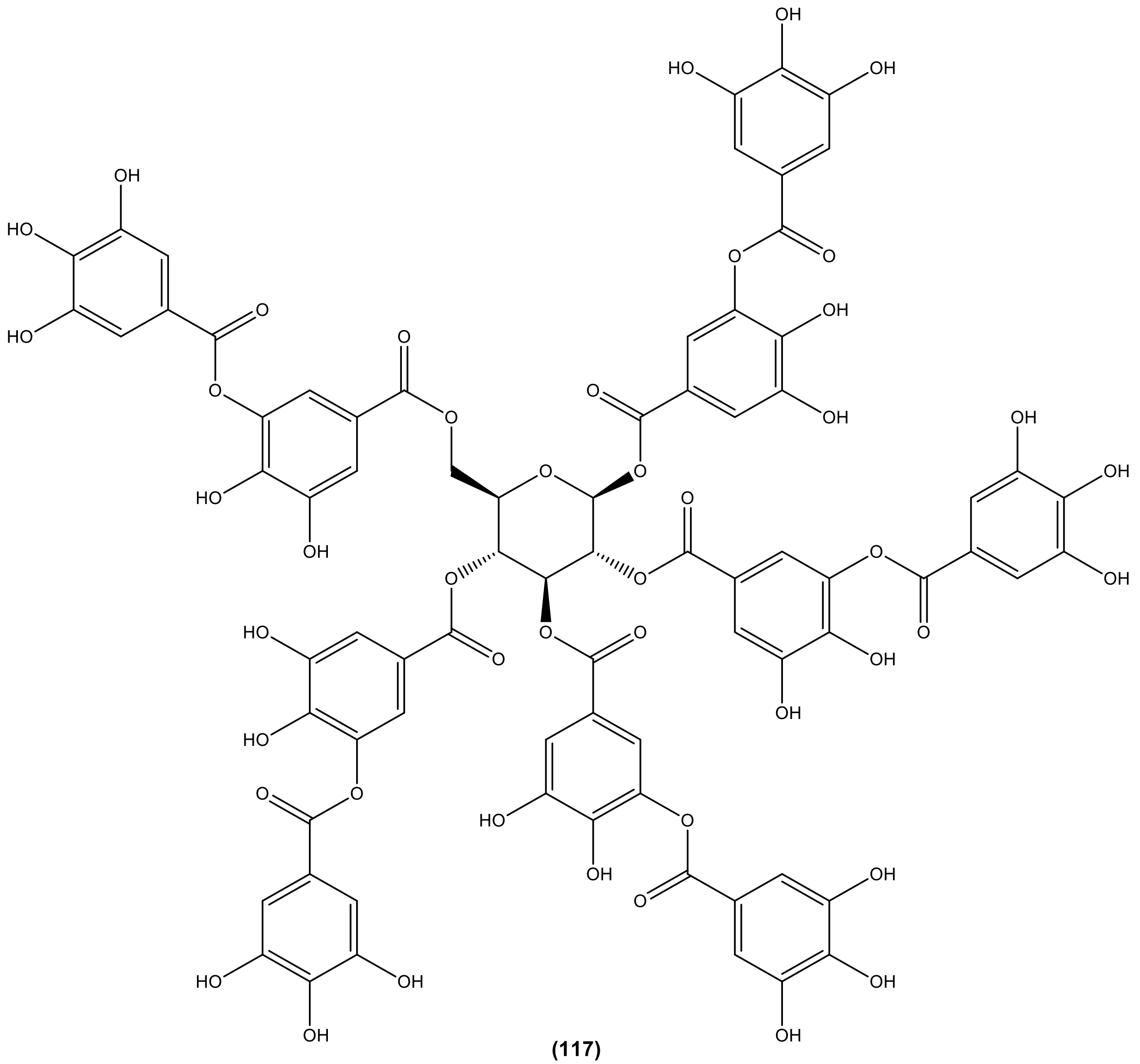
OH
HO
OH
O
O
OH
OH
HO
HO
O
O
O
O
O
O
HO
OH
OH
OH
OH
O
O
O
O
O
O
OH
O
HO
OH
O
OH
HO
O
HO
O
O
HO
OH
O
OH
O
HO
HO
OH
OH
OH
OH
OH

(117)

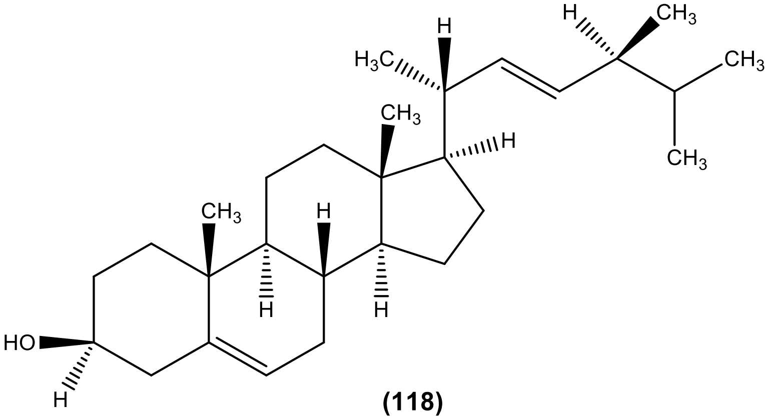

(118)

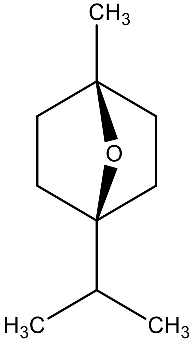

(119)

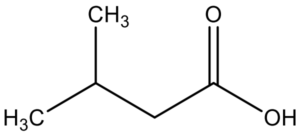

(120)

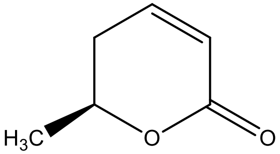

(121)

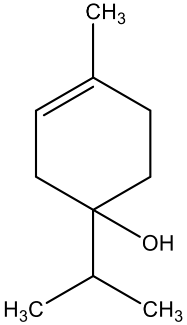

(122)

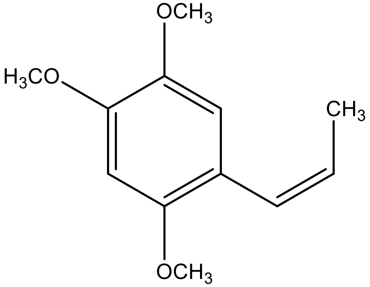

(123)

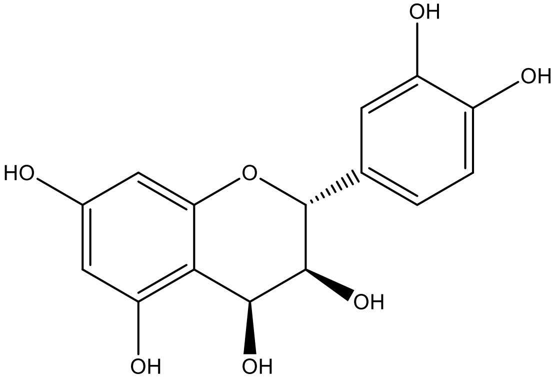

(124)

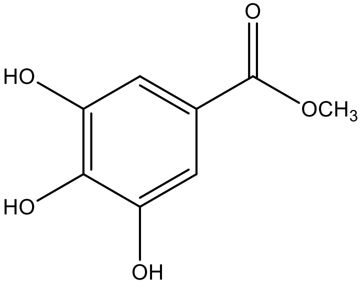

(125)

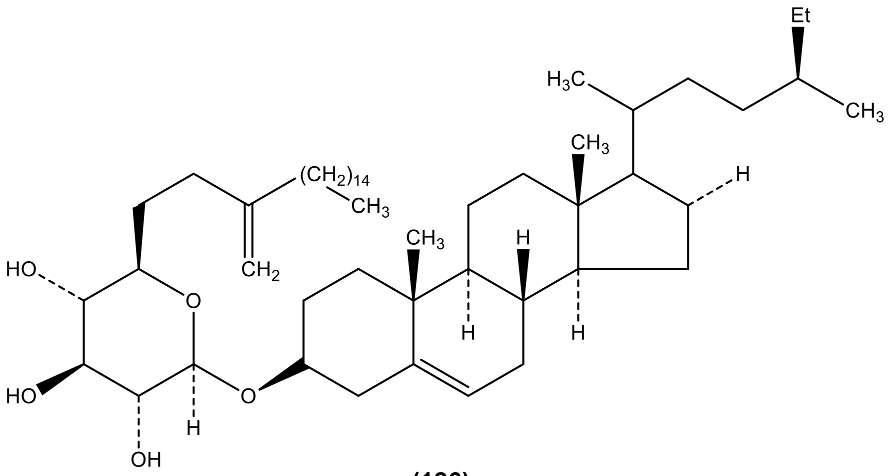

(126)

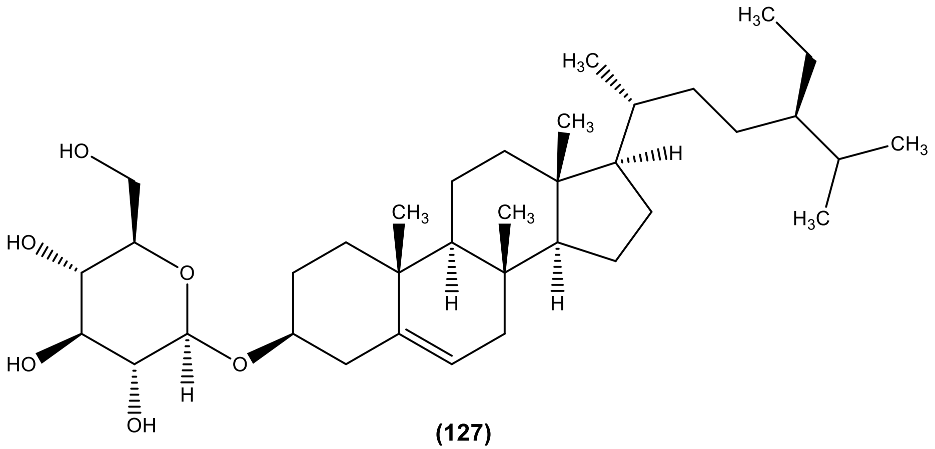

(127)

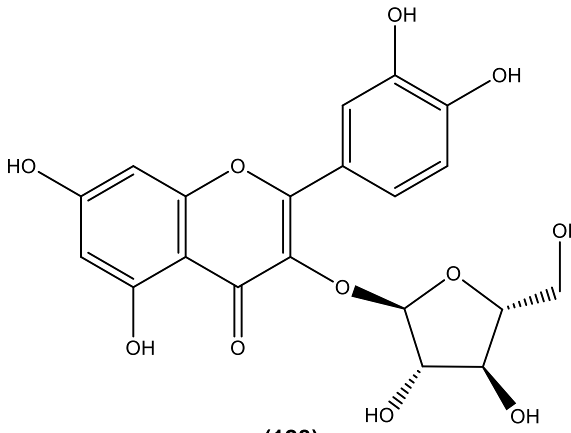

(128)

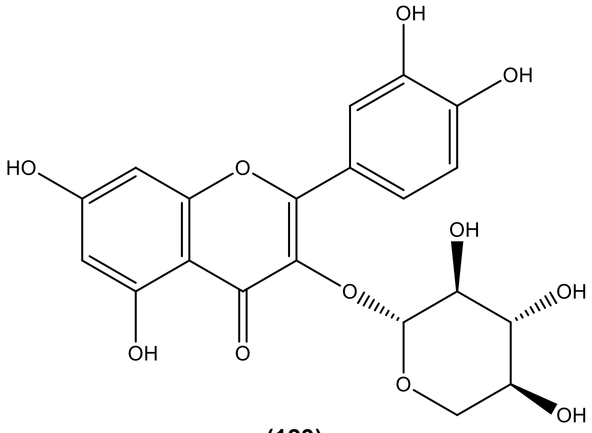

(129)

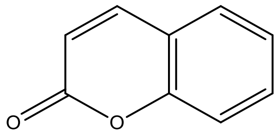

(130)

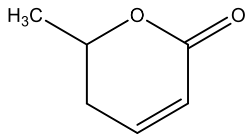

(131)

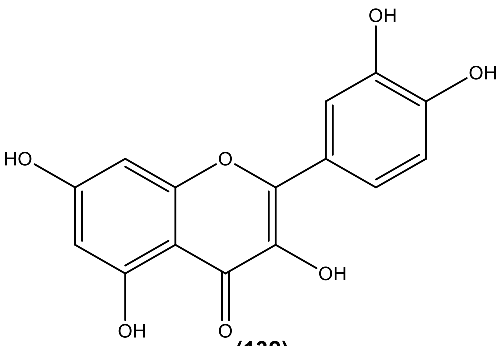

(132)

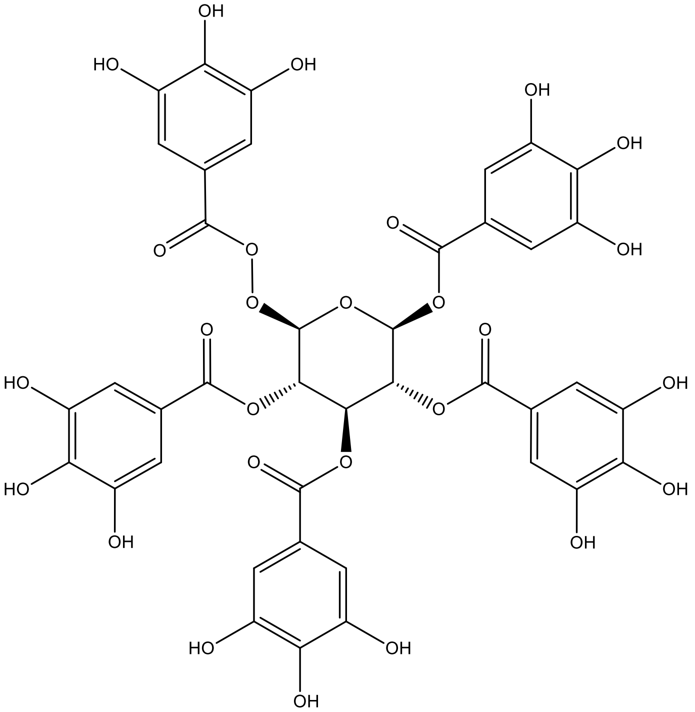

(133)

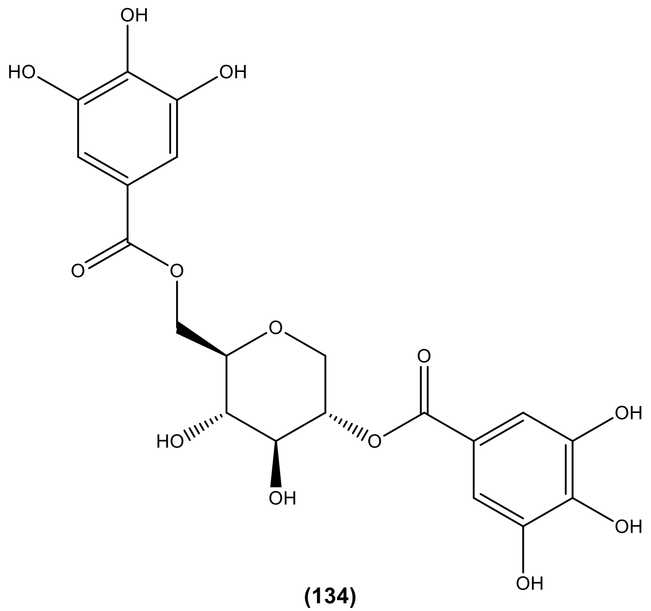

(134)

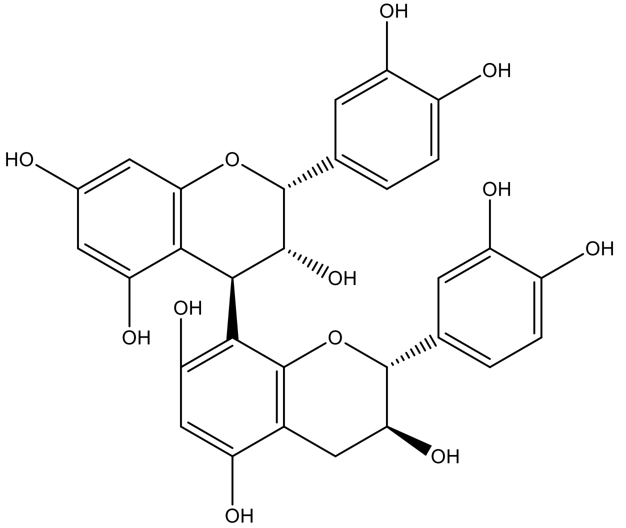

(135)

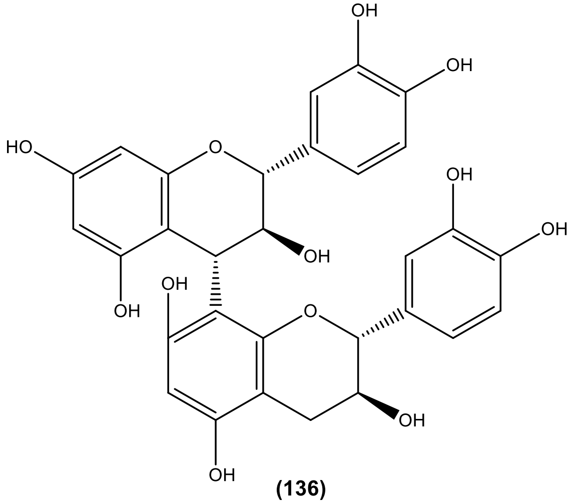

(136)

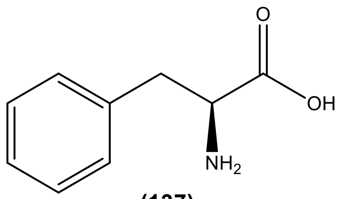

(137)

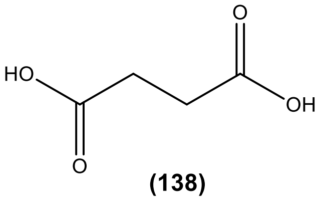

(138)

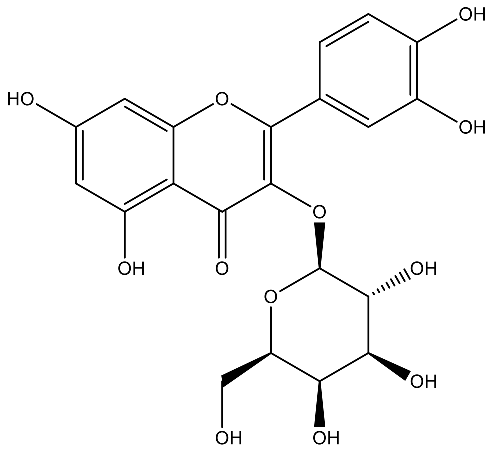

(139)

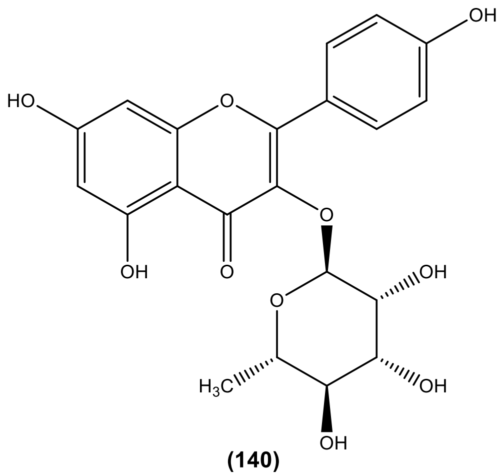

(140)

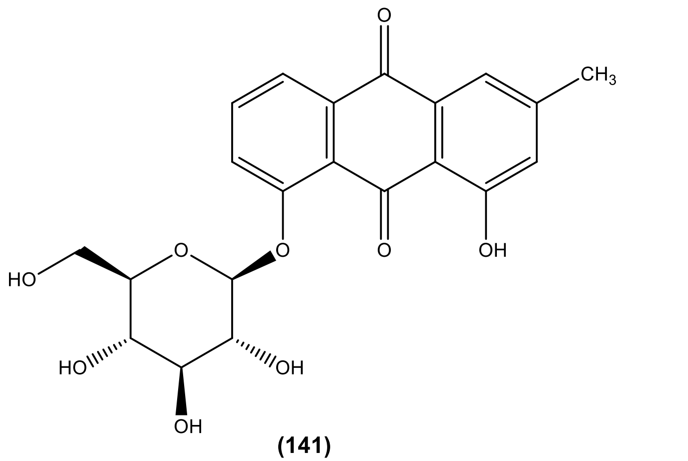

(141)

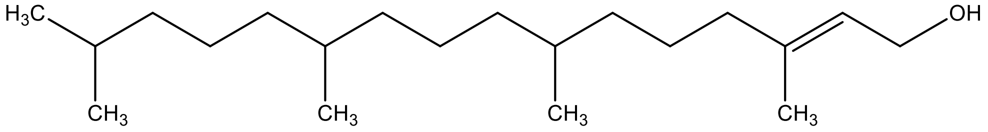

(142)

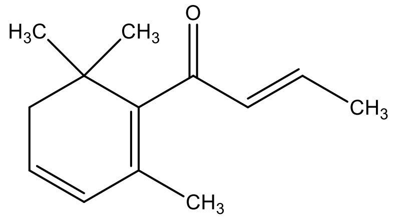

(143)

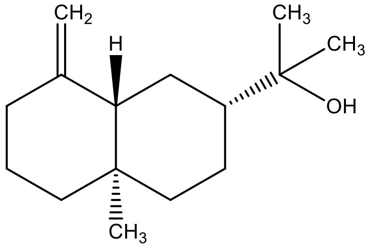

(144)

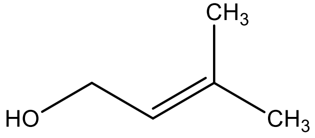

(145)

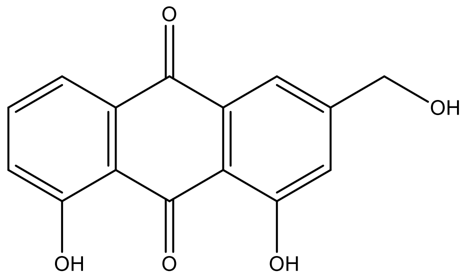

(146)

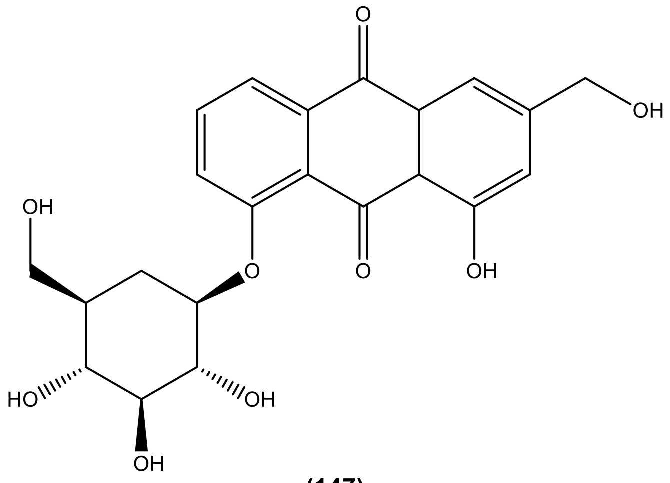

(147)

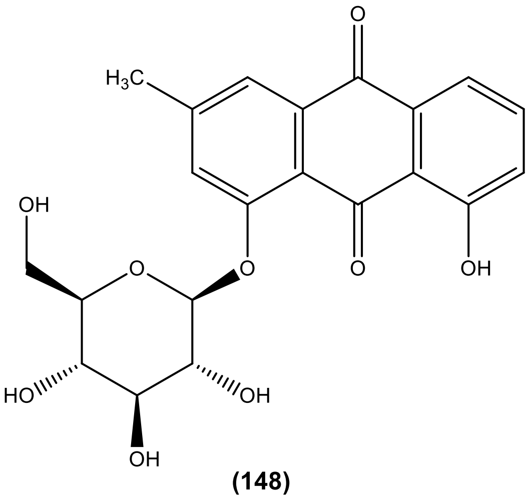

(148)

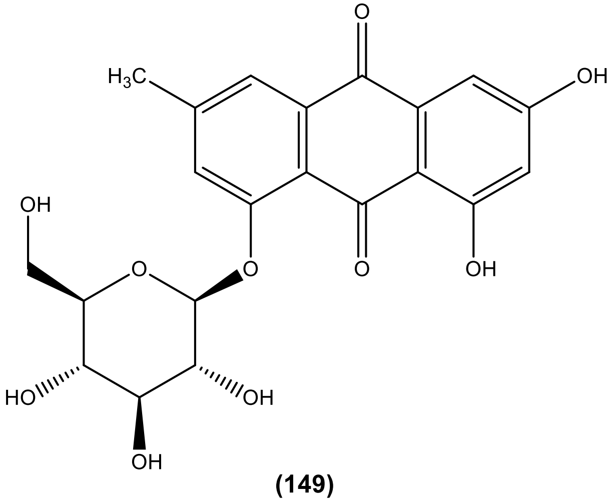

(149)

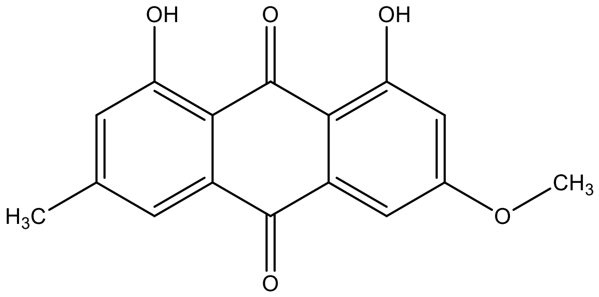

(150)

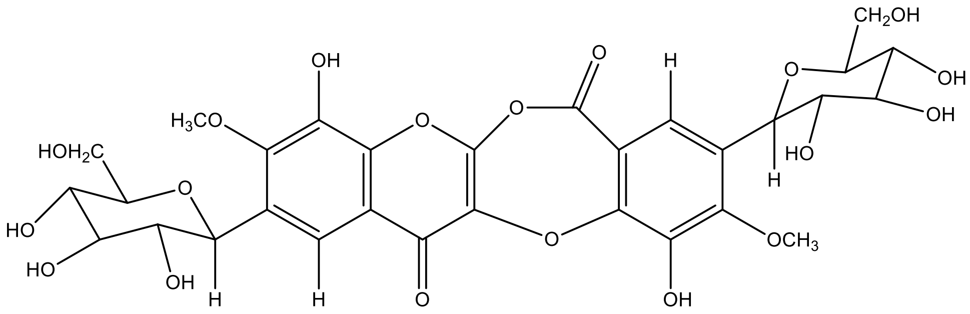
OH
H₃CO
HOH₂C
O
HO
HO
OH
H
H
O
O
O
O
O
H
CH₂OH
OH
OH
HO
H
OCH₃
OH

(151)

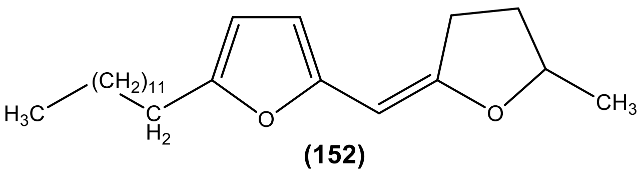
H₃C
(CH₂)₁₁
C
H₂
O
O
CH₃

(152)

Supplementary Table 1 Botanical description of *Bergenia* species

S.No.	Bergenia species	Botanical description	References
1	*Bergenia ciliata* (Haw.) Sternb.	It is commonly called as 'fringed bergenia', 'hairy-leaf bergenia' or 'winter begonia', is a large-leaved, slow-growing, clump-forming, perennial plant that normally grows to 12 inches tall and spreads to 24 inches wide. The plant grows as open rosettes with thick, leathery, finely-toothed, wavy-edged, broad, obovate to round 12-14 inches long green leaves. Leaves are pubescent (ciliate) on both the surfaces with toothed and fringed- margins. Flowers are pale pink with rose-pink calyces and bloom in spring season in clusters on 10 inches tall stout-stalks, above the foliage. In some climates, flowers appear before the emergence of new leaves.	[33]
2	*Bergenia crassifolia* (L.) Fritsch	commonly called 'leather bergenia', 'Siberian tea', 'Mongolian tea', 'winter glut', 'winter glow', 'winter blooming bergenia', 'heartleaf bergenia','elephant-ears' is a large-leaved evergreen perennial plant. Rosettes of leathery, fine-toothed, obovate-rounded green leaves (8 inches long and 7 inches wide) form dense, slowly-spreading clumps of 12 inches tall foliage. Lavender pink flowers bloom in panicles from March to early May atop rigid leafless stalks, rising to 18 inches tall.	[33]

		Flowers bloom in winter, hence the additional common name of ‘winter blooming Bergenia’. Although evergreen, the leaves of this plant often turn bronze and battered in cold winter temperatures.	
3	*Bergenia emeiensis* (C.Y. Wu ex J.T. Pan)	It is commonly known as ‘snow chimes’. It is a polycarpic plant that grows to a height of 35 cm and bears thick and scaly rhizomes. The leaves are petiolate, leathery and glabrous; cuneate at the base and obtuse at the apex. The inflorescence is cymose with sub-sessile, white or reddish flowers. The petals are narrowly obovate with claw-shaped base.	[31, 33]
4	*Bergenia ligulata* (Wall.) Engl.	It is commonly known as ‘Pashenbeda’ or ‘rockfoil’. It is a perennial herb that grows to a height of 35 cm. The root-stock of this plant is very stout and the stem is procumbent, thick and short. Rhizome is cylindrical, solid and barrel-shaped, 1-3 cm long and 1-2 cm in diameter with small roots. The stem bears few leaves that are glabrous or hirsute. The lamina is sub-orbicular to orbicular, with a cordate base and rounded apex while the margin is entire or denticulate. The petiole is glabrous or hirsute with sheathing towards the base. Flowers are pink to purple in colour contributing to one-sided raceme or corymbose inflorescence	[33]

5	*Bergenia purpurascens* (Hook. f. & Thomson) Engl.	It is commonly known as 'purple bergenia'. It is a perennial herb that grows up to a height of 13-50 cm and bears thick-scaly rhizomes. The plant exhibits basal phyllotaxy with 2-7 cm long petiole, obovate leathery-leaf blade (glabrous and glandular pitted on both-surfaces) with cuneate base and obtuse apex. Inflorescence is cymose, 3-23 cm long; branches and pedicels densely long glandular and hairy. Petals are purple and ovate with claw-shaped narrow-base.	[33, 34]
6	*Bergenia scopulosa* (T.P. Wang)	It is commonly known as 'elephant ear', 'Vuorenkilvet', 'Bergenie' and is a perennial herb that grows to a height of 10-50 cm and bears thick-scaly rhizomes. The plant exhibits basal phyllotaxy with 1.5-13 cm long petiole and leathery, glabrous, leaf blade having rounded base and obtuse apex and sinuate margin. The inflorescence is cymose and the petals are elliptic or broadly ovate with claw-shaped narrow base.	[33]
**7**	*Bergenia stracheyi* (Hook. f. & Thomson) Engl.	It is commonly known as 'Himalayan bergenia' and is a perennial herb that grows up to a height of 20 cm and bears thick rhizomes. The plant exhibits basal phyllotaxy with 1-1.8 cm long petiole and glabrous leaf blade that is obovate (base is cuneate and apex is obtuse). The flowers bear red petals with subspatulate base and obtuse apex, and contribute to cymose inflorescence.	[33]

8	*Bergenia hissarica* (A. Boriss)	*Bergenia hissarica* (A. Boriss) is an extremely rare, perennial, herbaceous, rhizomatous plant, endemic to Hissar Mountains. Leaves are basal, large, form rosette, oblong-obovate in shape. Leaf apex is obtuse and leaf-margins are densely covered with hairs. The flowers are bell-shaped; 6-8 flowers grow on each pedicel (devoid of glandular hairs) and blossom in May-June. Corolla consists of 5 whitish-pink or white petals. Rhizomes are strong, 1.0-2.5 cm thick, horizontal, covered with remnants of petioles of dead leaves. Fruit is a capsule that harbours elongated seeds. No records are available on the cultivation of *B. hissarica*.	[32]
9	*Bergenia tianquanesis* (J.T. Pan)	It is a perennial herb that grows up to a height of 25 cm and bears dark brown, thick and scaly rhizomes. The plant exhibits basal phyllotaxy with 1.5 cm long petiole and the leathery- glandular leaf blade is obovate (cuneate base and obtuse apex). Inflorescence is cymose type, The plant bears reddish flowers with petals that have claw-shaped narrow base.	[32,33]

Supplementary Table 2 Tissue culture reports of *Bergenia* species.

Species	Explant	Plant growth hormone used (mg/L)	Response	Reference(s)
	Leaf	MS media + BAP (0.0-2.0) + NAA (0.0-1.5)	Callus formation	
	Root tip	MS media + BAP (0.0-2.00) + NAA (0.0-1.5)	Failed to show any response	[169]
	Shoot tip	MS media + BAP (0.0-2.0) + NAA (0.0-1.5)	Failed to show any response	
	Node	MS media + BAP (3.0) + IAA (0.1) + AA (40)	Development of multiple shoots	[170]
B. ciliata	*in vitro* grown shoots	MS media + BAP (3.0) + IAA (0.1) + AA (40) + IAA (3.0)	Development of roots	
	Leaf	B5 ½ strength medium + TDZ (2) and NAA (2)	Callus formation	[171]
	Callus	B5 ½ strength medium + BAP (2) + IAA (1)	Development of multiple shoots	
	Leaf	MS media + NAA (0.50) + BAP (0.50) + dried hydrolyzed casein (100.00) + PVP (0.2%)	Callus formation	[172]
	Leaf	MS media + NAA (0.50) + IBA (0.10) + BAP (0.50)	Development of multiple shoots	
B. crassifolia	Leaf	1/2 MS media + IBA (0.50) + NAA (0.01)	Development of roots	
	Apical buds	MS media + NAA (0.1) + BAP (1.0) + PVP (0.5)	Development of multiple shoots	[97]
	in vitro grown shoots	½ MS media + IBA (1.0) + PVP (0.5)	Development of roots	
	Shoot tips	MS media + KN (10 μM) + IAA (7.5 μM)	Failed to show any response	
	Shoot tips	Gamborg's medium + KN (10 μM) + IAA (7.5 μM)	Development of multiple shoots	
B. ligulata	Shoot tips	Modified MS medium + KN (7.5 μM) + IAA (7.5 μM)	Development of shoots	
	Shoot tips	Nitsch and Nitsch + KN (10 μM) + IAA (7.5 μM)	Development of shoots	[173]
	Leaf	MS media + 2,4-D (0.2) + BAP (0.5) + NAA (0.4) + activated carbon (3000)	Formation of callus	
	Leaf	MS media + BAP (2.0) + NAA (0.2) + activated carbon (3000)	Development of shoot buds	
B. purpurascens	Leaf	MS + BAP (2.0) + NAA (0.2) + KN (2.0) + activated carbon (3000)	Development of multiple shoots	[174]
	In vitro grown shoots	½ MS + NAA (0.5) + activated carbon (3000)	Formation of roots	
